# In Situ/Operando Probing of Dynamic Phase Structures of Alumina‐Supported Ultrasmall Copper‐Gold Alloy Nanoparticles Under Reaction Conditions

**DOI:** 10.1002/anie.202508735

**Published:** 2025-06-16

**Authors:** Han‐Wen Cheng, Jing Li, Shiyao Shan, Xiaowei Lv, Guanyu Chen, Merry Madiou, Dong Dinh, Seyed Danial Mousavi, Zhi‐Peng Wu, Shan Wang, Yazan Maswadeh, Valeri Petkov, Susan Lu, Ke Pei, Wenbin You, Renchao Che, Chuan‐Jian Zhong

**Affiliations:** ^1^ Laboratory of Advanced Materials Shanghai Key Lab of Molecular Catalysis and Innovative Materials Department of Materials Science Academy for Engineering & Technology Fudan University Shanghai 200438 China; ^2^ Department of Chemistry State University of New York at Binghamton Binghamton NY 13902 USA; ^3^ Department of Physics Central Michigan University Mt. Pleasant MI 48859 USA

**Keywords:** Dynamic phase structures, Equiatomic copper‐gold alloy, In situ atomic‐scale morphological tracking, Operando ensemble‐averaged structural tracking, Ultrasmall nanoparticles

## Abstract

The ability to control phase structures and surface sites of ultrasmall alloy nanoparticles under reaction conditions is essential for preparing catalysts by design. This is, however, challenging due to limited understanding of the atomic‐scale phases and their correlation with the ensemble‐averaged structures and activities of catalysts during catalytic reactions. We reveal here a dynamic structural stability of alumina‐supported ultrasmall and equiatomic copper‐gold alloy nanoparticles under reaction conditions as a model system in the in situ/operando study. In situ atomic‐scale morphological tracking under oxygen reveals temperature‐dependent dynamic crystalline‐amorphous dual‐phase structures, showing dynamic stability over an elevated temperature range. This atomic‐scale dynamic phase stability coincides with a “conversion plateau” observed for carbon monoxide oxidation on the catalyst. It is substantiated by the stable lattice ordering/disordering structures and surface sites with oscillatory characteristics shown by operando ensemble‐average structural tracking of the catalyst during the oxidation reaction. The understanding of the atomic‐scale dynamic phase structures in correlation with the ensemble‐average dynamic ordering/disordering phase structures and surface sites provides fresh insights into the unique synergy of the supported alloy nanoparticles. This understanding has implications for the design and structural tuning of active and stable ultrasmall alloy catalysts under elevated temperatures.

## Graphical abstract

Supported ultrasmall copper‐gold alloy nanoparticles are active heterogenous catalysts. In situ TEM tracking under oxygen at different temperatures reveals atomic‐scale dynamic phase structures, whereas combined operando synchrotron HE‐XRD and DRIFTS characterizations uncover ensemble‐averaged ordering/disordering phase structures and oscillatory surface adsorbates, showing implications for designing highly stable ultrasmall alloy catalysts.

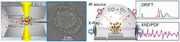

## Introduction

Catalytic conversions of carbon monoxide (CO) and hydrocarbons by molecular oxygen play a pivotal role in remediation of emission pollutants and production of sustainable energy. However, the instability of most metal catalysts under reaction conditions, especially the propensity of metal oxidation at elevated reaction temperatures, poses a key challenge in controlling the catalyst durability.^[^ ^1^, ^2^, ^3^, ^4^, ^5^, ^6^, ^7^, ^8^, ^9^, ^10^, ^11^, ^12^ ^]^ Driven by increasing concerns over environmental sustainability, there have been increasing studies of supported metal and metal oxide catalysts for CO and other hydrocarbons,^[^ ^7^ ^]^ focusing on investigating active sites in the reaction perimeter zones, active single atoms or nanoparticles (NPs),^[^ ^8^ ^]^ core‐shell/alloy phases, surface oxidized species,^[^ ^9^ ^]^ or intermediates^[^ ^10^, ^11^ ^]^ and lattice or vacancy oxygen species.^[^ ^11^, ^12^ ^]^ In particular, noble metal‐based catalysts for CO oxidation has attracted a great deal of interest partly because of CO being one of the major exhaust gases^[^ ^13^, ^14^, ^15^, ^16^ ^]^ and noble metal catalysts exhibiting low‐temperature oxidation activity.^[^ ^17^, ^18^ ^]^ As one of the most active noble metals for CO oxidation,^[^ ^19^, ^20^, ^21^ ^]^ Pt‐based catalysts have been extensively studied in terms of Pt particle size, composition, surface hydroxyl group, and support effects.^[^ ^22^, ^23^, ^24^, ^25^, ^26^ ^]^ In comparison, Haruta's breakthrough discovery of CO oxidation activity over supported Au NPs in the 3−5 nm size range has attracted widespread interest in Au‐based nanoparticle catalysts.^[^ ^7^, ^27^ ^]^ Indeed, nanoscale Au‐based catalysts for CO oxidation exhibit high activity, in contrast to the lower activity of bulk Au due to the filled d‐orbital electrons.^[^ ^28^, ^29^, ^30^ ^]^ Insights have been gained into the catalytic mechanisms of various noble metal catalysts for CO oxidation^[^ ^31^ ^]^ by using in situ characterization techniques such as ambient pressure X‐ray photoelectron spectroscopy, scanning tunneling microscopy, and surface X‐ray diffraction.^[^ ^32^, ^33^ ^]^ The strong electronic interactions between the support (e.g., Mg/Al ─ O) and Au NPs lead to the coexistence of Au^0^ and Au^δ+^ as active sites in activating lattice oxygen for partial HCHO oxidation.^[^ ^34^ ^]^ Studies of supported copper and copper‐gold (CuAu) catalysts have shown Au‐CuO perimeter zone activation^[^ ^35^, ^36^, ^37^, ^38^ ^]^ and phase segregation‐induced deactivation during CO oxidation.^[^ ^36^, ^38^ ^]^ However, fundamental questions on how metal‐support interaction, nanoscale alloying, Cu redox properties,^[^ ^3^ ^]^ and phase changes^[^ ^39^ ^]^ influence the catalytic activity under the reaction remain elusive. In an earlier study of CeO_2_‐supported AuCu NPs prepared by a contact residual method in which Au nanocrystals were pressed sequentially onto Cu (111) and CeO_2_ (111) surface,^[^ ^40^ ^]^ Au segregation occurs upon CO exposure, while Cu undergoes oxidization under O_2_. The Cu_2_O/AuCu interface facilitates CO─O interaction during CO oxidation. In a recent AP‐XPS study of highly‐ordered pyrolytic graphite (HOPG)‐supported CuAu NPs (8∼20 nm), surface segregation of Cu is shown to promote the dissociative adsorption of O_2_ and O spillover onto HOPG, leading to the formation of surface graphitic oxides to encapsulate CuAu NPs.^[^ ^41^ ^]^ Various ex situ and in situ techniques have been used in increasing studies of such systems,^[^ ^39^, ^40^, ^41^, ^42^, ^43^, ^44^ ^]^ leading to some mechanistic insights into the metal‐support interaction and oxygen‐induced atomic restructuring.^[^ ^44^ ^]^ Nevertheless, how the dynamic atomic‐scale morphology influences the surface sites and correlates with the ensemble‐average catalytic activities under reaction conditions remains one of the most challenging questions. This challenge stems from some of the catalyst or system‐related complexities in real‐time tracking of the nanoscale catalysts, especially for sizes down to sub‐5 nm (ultrasmall) and multiple metallic/alloy compositions.

We show herein the real‐time atomic‐scale tracking of phase dynamics of ultrasmall (<5 nm) alloy CuAu/γ‐Al_2_O_3_ catalysts under oxygen using in situ environmental transmission electron microscopy (ETEM), which distinctively differs from most of the previous studies of catalytic oxidation over >10‐nm AuCu NPs on other supports. Importantly, novel insights are unraveled into the correlation between the atomic‐scale phase dynamics, and the ensemble‐averaged dynamic phase structures and surface species under reaction conditions. The latter was determined by combined operando diffuse reflectance infrared Fourier transform spectroscopy (DRIFTS) and synchrotron high‐energy X‐ray diffraction (HE‐XRD).^[^ ^45^ ^]^ In this in situ/operando study, we chose equiatomic CuAu alloy NPs in ultrasmall sizes as a model system for three reasons. First, this bimetallic composition is documented as a disordered solid solution, and is known for gold brazing alloys in creating strong, ductile braze joints in applications in metallized ceramics, ceramic to metal seals, and glass to metal seals. Second, for equiatomic CuAu alloys in macroscopic deformation and shape restoration, the random structure leads to a disordered fcc solid solution,^[^ ^46^ ^]^ whereas the ordering transition induces a significant change from fcc (Fm‐3 m with a = 0.389 nm) to fct (P4/mmm with a = 0.397, c = 0.367 nm),^[^ ^47^ ^]^ involving a tetragonal distortion of the fcc lattice in L1_2_ (Cu_3_Au and Au_3_Cu, Pm‐3 m) structures^[^ ^48^ ^]^ (Figure S1). Third, alloying Cu with Au allows the protection against oxidation into the bulk. This was shown by experimental and theoretical studies of hyperthermal O_2_ oxidation of CuAu alloy surfaces, revealing segregation of Au NPs to the top layer. CuAu(111) and Au_3_Cu(111) exhibit less susceptibility to oxidation than Cu_3_Au(111).^[^ ^49^ ^]^ Although the steady‐state structural ordering‐disordering is known to play a role in catalytic oxidation reactions,^[^ ^2^, ^6^, ^35^ ^]^ little is known about how dynamic ordering‐disordering under oxygen influences catalytic oxidation at different temperatures. A key unanswered question is how the chemically ordered and disordered fcc phase structures occur at both atomic scales and ensemble‐averaged levels in correlation with catalytic activity and stability. We demonstrate here that new mechanistic insights can be gained by in situ TEM probing of the atomic‐scale dynamic phase structures of supported CuAu nanoparticles under reaction conditions, which is recently shown to be effective for other alloy nanoparticles,^[^ ^50^ ^]^ in correlation with the ensemble‐averaged structure changes determined by operando HE‐XRD and DRIFTS characterizations.

## Results and Discussion

CuAu NPs of different compositions were synthesized in aqueous solution by varying feeding ratios of HAuCl_4_ and Cu(NO_3_)_2_ in different precursor concentrations. Sodium acrylates were used as both reducing and capping agents at room temperature in a modified wet‐chemical synthesis protocol under N_2_ (Supporting Information).^[^ ^51^ ^]^ The as‐synthesized CuAu NPs could be transferred to organic phase by organothiol ligands, which exhibit particle morphology similar to those synthesized directly in organic phase.^[^ ^2^ ^]^ We focused on equiatomic CuAu NPs in this work in terms of the ultrasmall size and uniform bimetallic composition (Cu_50_Au_50_ NPs, 3.4 ± 0.4 nm, Figure S2). A key novel aspect of our approach to the preparation of the ultrasmall NPs was the post‐synthesis phase transfer and separation for subsequent dispersion on γ‐Al_2_O_3_ support. The post‐synthesis phase transfer of the as‐synthesized NPs in aqueous solution involved ligand exchange reaction with dodecanethiols in hexane phase. The resulting NPs were then supported onto alumina (see Figure S3, and Method in Supporting Information). The CuAu/γ‐Al_2_O_3_ catalysts were thermochemically treated under 20% O_2_ at 250 °C to remove the capping molecules and then calcined under 15% H_2_ at 400 °C. The particle size, size distribution, bimetallic composition, and alloy structure of both the as‐prepared and γ‐Al_2_O_3_‐supported Cu_50_Au_50_ NPs catalysts were characterized by TEM, XRD, and EELS (see Supporting Information).

**Figure 1:**
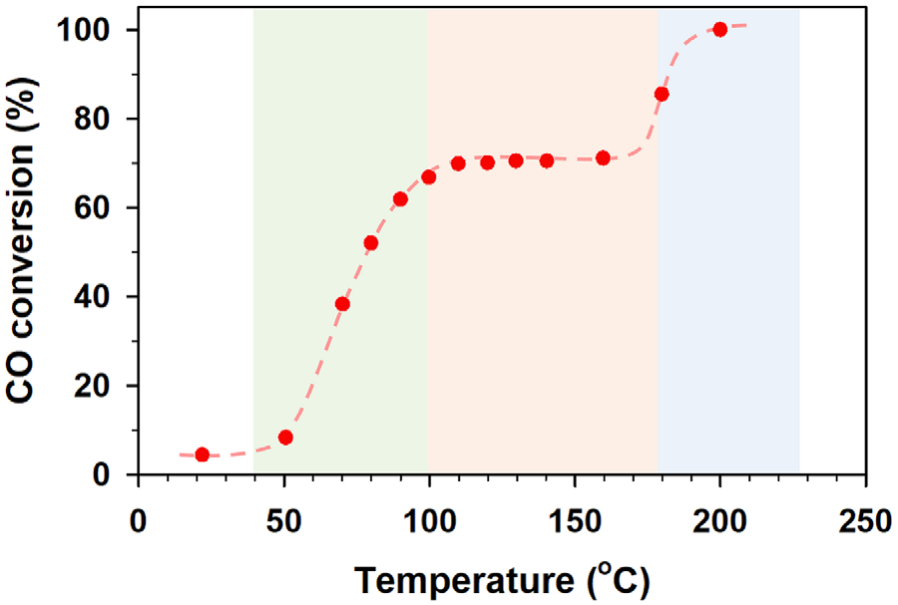
CO conversion versus reaction temperature over Cu_50_Au_50_/Al_2_O_3_ catalyst, showing a “conversion plateau” (CP) in the temperature range of 100°C∼170 °C. The colored regions highlight the temperature‐independent CO conversion, that is, CP, between the two temperature‐dependent CO conversions before and after the plateau.

As shown in Figure 1 for CO oxidation over Cu_50_Au_50_/Al_2_O_3_ catalyst, a “conversion plateau” (“CP”) is revealed in the temperature range of 100°C–170 °C. This surprising observation contrasts with conventional CO conversion‐temperature curves known for CO oxidation by O_2_ over CuAu catalysts with different compositions.^[^ ^2^, ^35^, ^52^ ^]^ In this observation, there are three stages in terms of the reaction temperature: i) an initial gradual increase at < 100 °C; ii) an activity plateau (67% conversion) at 100°C–170 °C; and iii) a subsequent gradual increase at >170 °C. The value of *T*_50_ (the temperature at 50% CO conversion) is about 78 °C.

The span of CP over a ∼70 °C range is, in fact, reproducible, as evidenced by several cycles of the measurement (Figure S4). It differs from the “hysteresis platform” observed for CO oxidation over Au/CuO/Al_2_O_3_ prepared by traditional impregnation where the activity increases and decays over ∼25 °C span due to a dynamic CO adsorption between Au and CuO active sites.^[^ ^52^ ^]^ To understand the mechanistic details of CP and determine if dynamic structure enables the stabilization of activity over the indicated temperature range, we carried out an in situ ETEM study of atomic‐scale dynamic morphologies of Cu_50_Au_50_/Al_2_O_3_ under O_2_ at different temperatures in correlation with ensemble‐averaged phase structures and surface‐active sites from operando XRD and DRIFTS characterizations.

### Atomic‐Scale Dynamic Morphology

**Figure 2:**
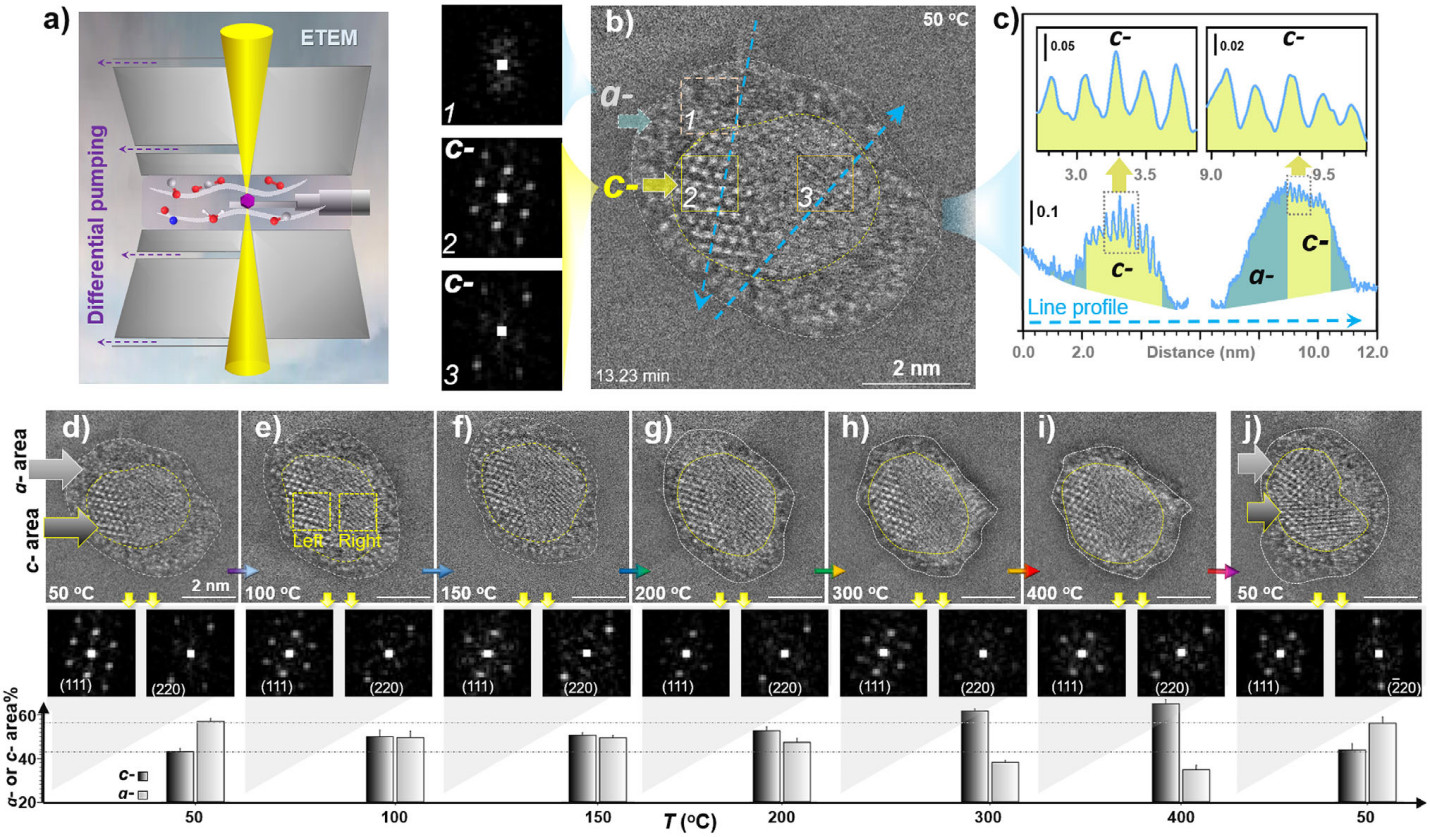
In situ ETEM (a) tracking of the temperature dependence of the atomic phases of γ‐Al_2_O_3_‐supported CuAu NP (with an initial *c‐*size: 3.9 nm, see Figure S5) under O_2_ (20 Pa) at different temperatures ranging from 50 (b–d) to 400 °C (e–i) and back to 50 °C (j). c) The corresponding line intensity profiles (see the two dashed blue arrows in b), showing the lattice spacing features for the two based areas (2 and 3) in the *c*‐phase (top row) and the contrast between the *c‐* and *a‐* phases (bottom row). Top panel: the TEM images (scale bars: 2.0 nm); middle panel: FFT patterns (as illustrated by left and right dash‐box in image); and bottom panel: percentages of the *a*‐phase (light grey) and *c*‐phase (dark grey) areas projected on the support surface versus temperature.

Figure 2a shows a representative in situ TEM image of Cu_50_Au_50_ NP on Al_2_O_3_ under O_2_ at 50 °C. The NP exhibits a crystalline (*c‐*) phase surrounded by an amorphous (*a‐*) phase (Figure 2b). The *c*‐phase appears to show a subtle indication of twining crystalline features between boxed regions 2 and 3, but the boundary is not apparent due to amorphization. This is evidenced by line profiles and lattice spacing features (Figure 2c). While the *a*‐phase around the *c*‐phase shows a spreading‐like feature on the support surface, the *c*‐phase shows two apparent morphological domains as revealed by FFT analysis (Figure 2b insets). To understand the dynamic domain structures, the atomic‐scale details of the supported NP were tracked in a temperature range of 50°C–400 °C (Figure 2d–i), revealing subtle evolution for the crystal twining and the *a*‐/*c*‐phase structure changing. This dynamic biphasic characteristic exhibits an apparent temperature‐dependent reversibility in terms of the relative biphasic percentage upon changing the temperature from 50°C to 400 °C (d–i) and back to 50 °C (j). By changing the temperature from 50°C to 400 °C, the gradual increase in the percentage of *c*‐phase is accompanied by a decrease in the percentage of *a*‐phase, which is reversed upon cooling back to 50 °C. This observation reflects morphological evolution of the supported NP due to differences in the NP‐support interaction at different temperatures. For the oxidized copper species, CuO appears at all temperatures (<300 °C). At >300 °C, it is possible that an increased amount of CuO phase is transformed to Cu_2_O phase. While the detailed distribution of the phases may be too complex to determine the structures, preliminary analysis was suggestive of CuO–Cu_2_O phase transformation with a possible intermediated species (Cu_4_O_3_ phase). Surprisingly, the dual‐phase evolution at 100°C–150 °C reveals a constant *a*‐/*c*‐ phase ratio, where several different phases can be identified, including CuAu (fct), Cu_3_Au (Pm‐3 m), Au_3_Cu (Pm‐3 m), Au, and Cu_2_O. This temperature range coincides with that corresponding to the “CP” phenomenon shown in Figure 1. The fact that the relative proportion of the two phases changes while the overall NP's morphology remains unchanged suggests the possibility of dynamic stability of the phase structures within this temperature range, in comparison with other supported alloy nanoparticles.^[^ ^50^ ^]^ Taken together from the observation in Figure 2b and d–j and the analysis based on lattice and FFT pattern, these phase structures likely consist of a mixture of CuAu (fcc/fct) and CuAu_3_ (ordered‐fcc, Pm‐3 m) phases, as shown by left domain of the NP, and a mixture of Cu_3_Au (Pm‐3 m) and Cu_2_O/Cu_4_O_3_/CuO phases,^[^ ^35^, ^44^, ^49^, ^53^, ^54^, ^55^, ^56^, ^57^, ^58^ ^]^ as indicated by the right domain. The identification of the crystalline or amorphous is not only substantiated by analyzing these images’ intensity but also by measurement of the sample in different orientations, showing sharp contrast among the *c*‐phase, *c*‐/*a*‐phase boundary, and the *c*‐/*a*‐phase transformation over the support in terms of crystallinity and reversibility. As shown by analysis of the FFT patterns obtained from the two domains of *c‐*phase in Figure 2 (see also Figure S5), (111) and (220) planes for CuAu (fct), Cu_3_Au (Pm‐3 m) and Cu_2_O are identified in [‐110] direction (Figure S6).

**Figure 3:**
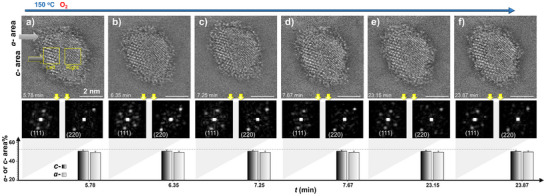
In situ TEM images of γ‐Al_2_O_3_‐supported CuAu NP (same NP as in Figure 2) under O_2_ at 150 °C (see also Figure S7 for 100 °C) as a function of time (The exact imaging times from (a) to (f) are indicated at the lower left of the TEM images). Top panel: the TEM images (scale bars: 2.0 nm); middle panel: FFT patterns (as illustrated by left and right dash‐box in image); and bottom panel: percentages of the *a*‐phase and *c*‐phase areas projected on the support surface versus time (see Figure S8 for line profile).

The dynamic stability of the supported NP is further tracked isothermally. As shown by in situ tracking images at 150 °C in Figure 3, the NP maintains the relative percentages of *a*‐ and *c*‐phases while the overall phase structures appear to exhibit subtle oscillatory variations among mixed phases, including CuAu (fct), Cu_3_Au (Pm‐3 m), Au_3_Cu (Pm‐3 m), Au, and Cu_2_O/CuO phases. This is indicative of a remarkable morphological stability considering the coexistence of the multiple phase structures. The *a*‐/*c*‐ phase ratio remains basically constant, demonstrating again the dual‐phase stability at the specific temperature range.

Upon switching the exposure from O_2_ to H_2_ in the in situ TEM tracking experiment, the dual‐phase is transformed to *c*‐phase featuring fcc‐CuAu phase (Figure S9), revealing a subtle time‐ and temperature‐dependent oscillatory characteristic in terms of the specific phase structures. Under H_2_, most of the NP exists as reduced CuAu. Similar biphasic characteristics are also observed with a 5‐nm sized CuAu/Al_2_O_3_ under O_2_ (a) and H_2_ (b) at different temperatures (Figure S10), which shows a much less degree of the dual phase evolution under O_2_. The size dependence is demonstrated by tracking NPs with small and large sizes in the same imaging frames, showing a greater degree of dynamic phase evolution for small NPs. The dynamic evolution of the ultrasmall NPs apparently depends on the NP‐support and NP─NP interactions. There is a clear contrast between the dynamic co‐existence of *a*‐ and *c*‐phases in the ultrasmall NPs and the attenuated co‐existence in larger NPs. This finding may have implications for studies of size‐dependent catalytic activity in supported metal/alloy NPs. While the increased surface area to volume ratio and higher proportion of surface atoms would make smaller NPs to exhibit a higher catalytic activity, this general expectation depends on a combination of size and closely related factors. For the supported CuAu NPs, some of these factors include phase dynamics, bimetallic synergies, and surface structures. Note that the size‐dependent catalytic activities for CO oxidation are mostly reported for unary NPs such as Pt, Au, Cu, including ultrasmall sizes.^[^ ^1^, ^3^, ^7^, ^31^ ^]^ Little or none is reported in terms of pure size effect on the activity for CuAu NPs,^[^ ^36^, ^38^ ^]^ including earlier studies of catalysts with small‐sized CuAu NPs on TiO_2_, SiO_2_, or Al_2_O_3_.^[^ ^59^ ^]^ The difficulty to resolve size effect for CuAu NPs stems from the complex operation of particle size, bimetallic composition, and phase structure in comparison with unary Au or Cu NPs.

**Figure 4:**
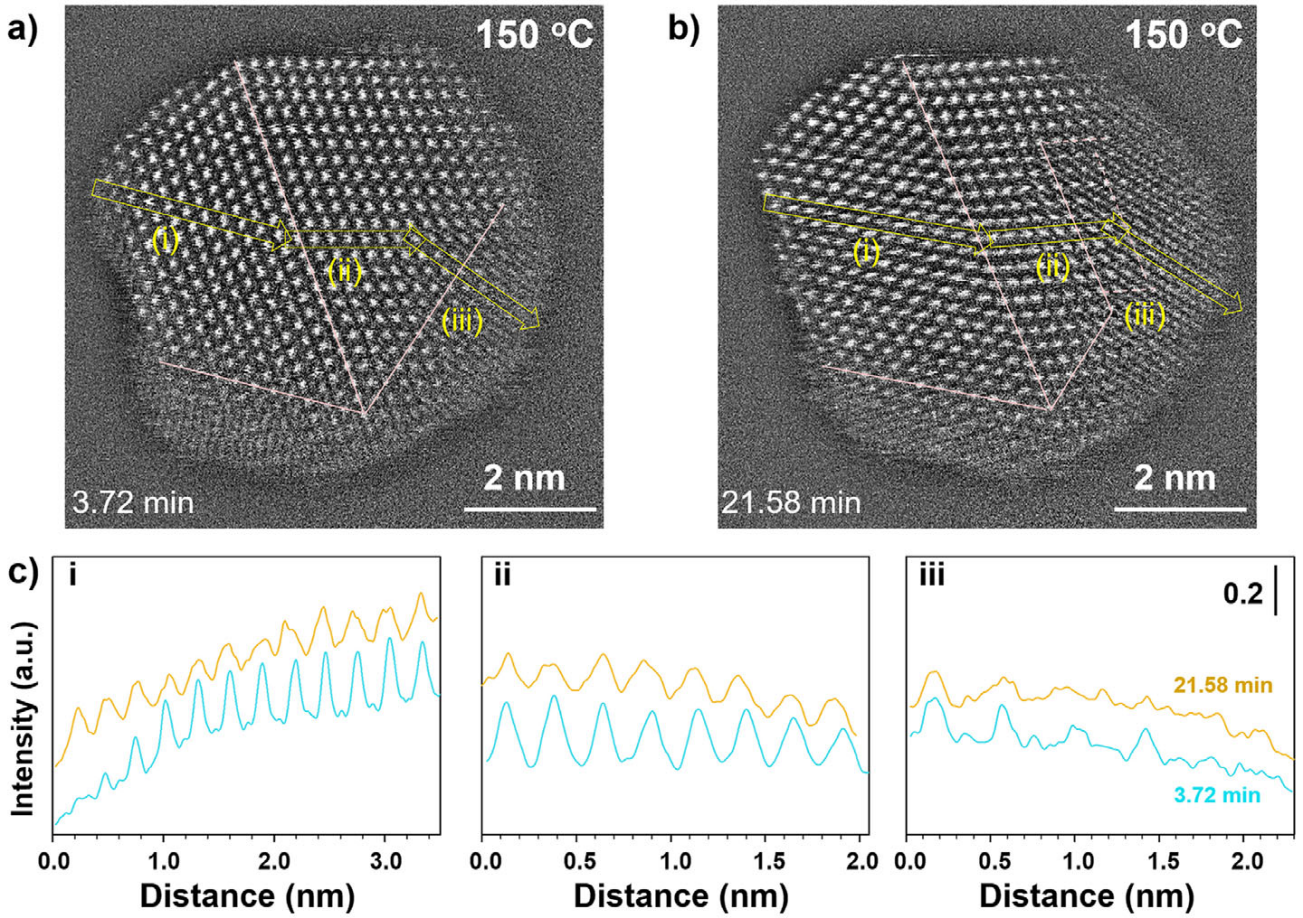
In situ TEM tracking of a 6.5 nm NP under O_2_ (20 Pa, 150 °C). a,b) TEM images (see FFT patterns in Figure S12) from 3.72 min (a) to 21.58 min (b). The lines in images illustrate crystalline twinning boundaries. c) Bottom panel showing line profiles in three regions from the left to the right side of the NP (i, ii, and iii), as illustrated by arrows in HAADF images (a,b).

The apparent dynamic stability of supported ultrasmall NPs under O_2_ is complex, involving a combination of fcc/fct phase transformation, CuO_x_ formation, and *a/c* dual‐phase evolution (∼3.9 nm, Figures 2, 3). To gain an insight into the atomic‐scale details, we examined a larger‐sized NP which offers a higher degree of crystallinity. As shown in Figure 4 (and Figure S11) for a supported CuAu NP (e.g., 6.5 nm), where a multiple twin crystalline feature is evident from the left side to the right side of the NP, there is an apparent dynamic evolution. Clear contrasts are observed in three regions of the *c*‐phases, upper‐left region (i), middle region (ii), and lower‐right region (iii) (Figures 4a,b and S12), reflecting different degrees of AuCu and CuO_x_ at different times. These changes are further substantiated by the cross‐sectional line profiles in terms of interatomic spacing, indicative of the presence of oxygen in the lattice (iii) (Figures 4c and S11). As an estimate of the apparent mobility of lattice/vacancy/interstitial oxygen species (O_L/V/I_),^[^ ^60^ ^]^ we analyzed the in situ TEM images from tracking the NP under H_2_ where the oxygenated NP is reduced by H_2_ to a certain degree depending on the temperature. A rough estimate yielded a diffusion coefficient of ∼2 × 10^−17^ cm^2^ s^−1^ for the dynamic oxygen and vacancy in the lattice structure, which is comparable to those reported for lattice/vacancy oxygen in metal oxides.^[^ ^61^ ^]^ On one hand, the comparison of the patterns among regions (i, ii, and iii) reveals differences in line intensity, which correlates with the particle's thickness (or NP's height or size) passed through by the elastic scattered electrons.^[^ ^62^ ^]^ In 3D morphological visualization through analyzing the intensity correlation with the NP thickness,^[^ ^63^, ^64^ ^]^ the signal intensity (*I*) depends on the elastic scattering of electrons by the atoms in a linear relationship (*I* = *I*_sub_ + *αh*, where *I*_sub_ is the intensity scattered by the substrate, *h* is the NP thickness or pass length under the electron beam, and *α* is related to the imaging condition and the materials properties). This relationship, however, does not fully explain the intensity change in region‐iii. On the other hand, the observation of twin boundaries or multiply‐twinned crystalline features from region i to ii and in region iii, which is common in AuCu NPs,^[^ ^57^ ^]^ and their changes, serve as an indication of the dynamic crystallinity of the NP interacting with the support. This dynamic small crystal twinning also provides useful information for assessing the observed *a*‐/*c‐*phase dynamics of the ultrasmall NPs. Twinning occurs during phase transformations due to mismatch of atomic structures of Au and Cu. The presence of small twin crystals oriented with respect to each other could lead to amorphization by such mismatch induced high strains or defects. The release of the strains leads to the formation of disordered amorphous domains.^[^ ^65^ ^]^ Such amorphization can occur at the twin boundary or within the surrounding region, which is much pronounced for ultrasmall AuCu NPs.

The linkage of the dynamic evolution of the lattice/vacancy/interstitial oxygen species during the *a*‐/*c*‐phase transformation to oxygenation is supported by the results obtained from H_2_‐TPR (Figure S13) and CO‐TPR characterization of the oxygenated CuAu/Al_2_O_3_ (Figure S14). A key indication is that the amount of surface/subsurface oxygen species (O*) clearly scale with the oxygenation temperature and time, as well as the bimetallic composition. For example, by oxygenation treatment of the catalyst at 150 °C, the oxidized components can be reduced by H_2_ at different temperatures. This is shown by observing different peaks in the H_2_‐TPR curves, which reflect different CuO_x_ species (e.g., ∼193.1 °C, CuO; ∼283.4 °C, Cu_4_O_3_; ∼363.1 °C Cu_2_O; and ∼459.1 °C Cu_3_O_4_) (Figure S13). In general, while the reduction potential by hydrogen follows the order CuO > Cu_4_O_3_ > Cu_2_O > Cu_3_O_4_, the reduction temperatures exhibit the order CuO (200°C–250 °C) < Cu_2_O (250°C–450 °C). The intermediate phases (Cu_3_O_4_ or Cu_4_O_3_) fall in between. By correlating the relative abundance of the CuO_x_ species with the temperature, we believe Cu(I) species likely play a significant role in the catalytic synergy, which is consistent with the assessment that Cu(I) has a higher catalytic activity for CO oxidation than Cu(II).^[^ ^66^ ^]^ Moreover, the formation of a protective Cu_2_O layer can inhibit further oxidation to CuO, which allows the co‐existence of CuAu alloy under O_2_ at intermediate temperature.

**Figure 5:**
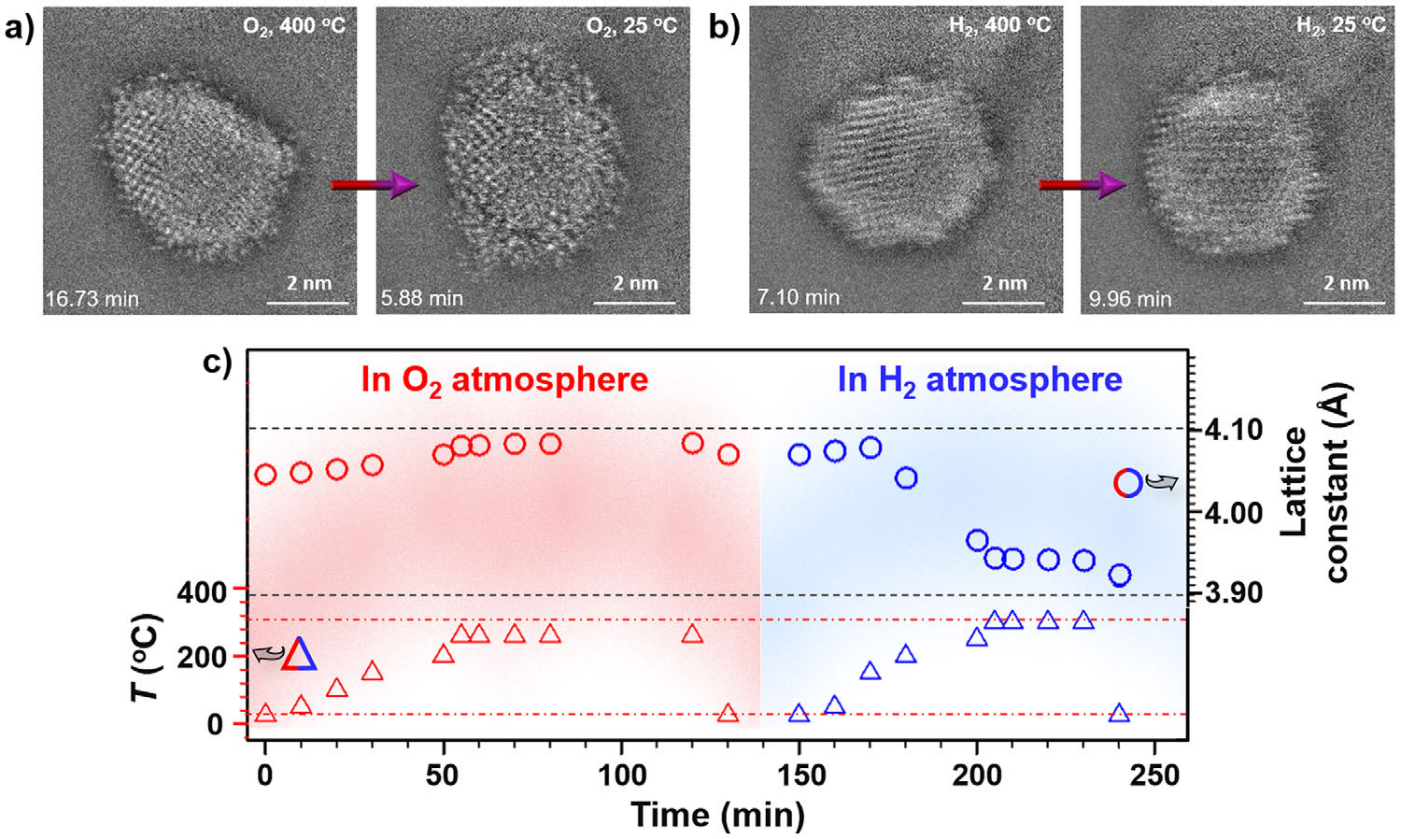
Morphology and structure changes under O_2_ and H_2_. a,b) In situ TEM images of the same NP shown in Figures 2, 3 under O_2_ (a) and H_2_ (b) at 400 °C and 25 °C, respectively. c) Lattice parameters (based on fcc‐type structure) extracted from in situ HE‐XRD/PDFs data for Au_51_Cu_49_/C under 10 vol.% O_2_, followed by 5 vol.% H_2_ at the different reaction temperatures (see Figure S16 for the in situ HE‐XRD pattens).

The oxygenated phase structure is also assessed by comparing the in situ TEM images under O_2_ and H_2_ atmosphere. A representative set of images is shown in Figure 5a,b for CuAu/Al_2_O_3_ first under O_2_ (a), and subsequently under H_2_ (b). Under O_2_, the mostly *c*‐phase NP exhibits a slight *a*‐phase along edges at 400 °C and is transformed into *a*‐/*c*‐phase morphology at 25 °C (a). There is an apparent indication of the presence of Cu_2_O/CuO phases (by FFT analysis in Figure S15). Subsequently, under H_2_, the NP displays crystalline CuAu fcc/fct phases with significantly diminished CuO_x_ phases, demonstrating clear reducibility of these oxide phases under the in situ condition.

This redox characteristic is further substantiated by in situ synchrotron HE‐XRD characterization coupled with pair distribution function (PDF) analysis (HE‐XRD/PDF) to determine the evolution of the lattice parameter under O_2_ and H_2_ at different temperatures (Figure 5c). HE‐XRD/PDF differs from regular in‐house XRD^[^ ^53^, ^54^, ^55^, ^56^, ^57^ ^]^ by its abilities to have a higher penetration into the sample and analyze both crystalline and amorphous structures, the latter of which is particularly important for characterizing ultrasmall (< 5 nm) nanoparticles. Carbon‐supported CuAu NPs (CuAu/C) were used in this experiment to minimize the possible contribution from the Al_2_O_3_ support. The fcc‐type lattice parameter is shown to increase with temperature under O_2_, which remains largely “increased” even after cooling down to room RT. In contrast, the lattice parameter decreases with increasing temperature under H_2_, and remains “shrunk” after being cooled down to room RT. The observed lattice expansion/shrinking is consistent with oxygenation/deoxygenation in the lattice structure.^[^ ^67^ ^]^ Importantly, the persistent fcc‐type structures throughout the oxidation and reduction processes demonstrate nanophase stability and structural integrity, even after deep oxygenation/deoxygenation of the NPs.

### Correlation with Ensemble‐Averaged Lattice and Surface Species Under Reaction Conditions

**Figure 6:**
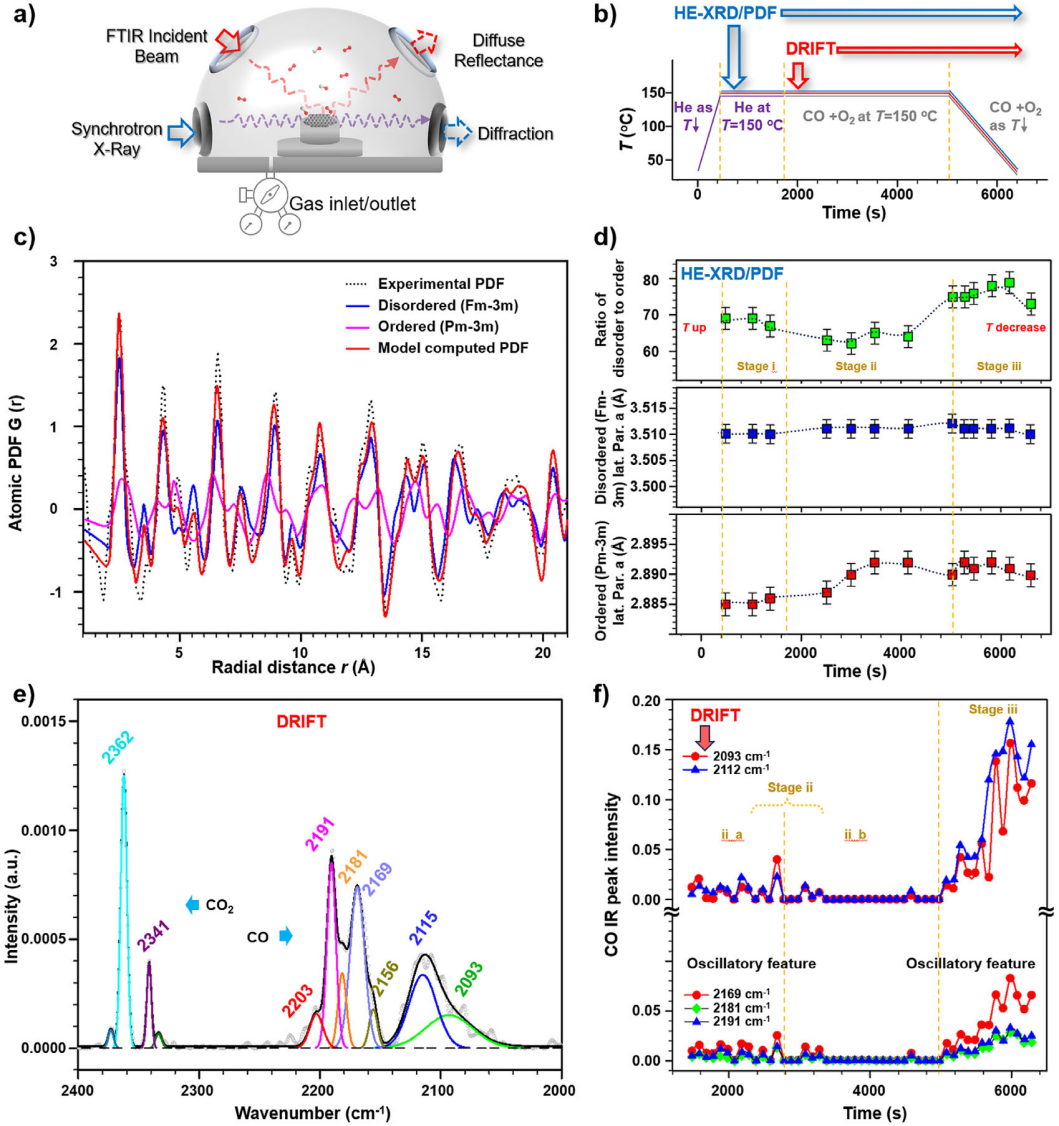
Combined in situ HE‐XRD/PDF and DRIFTS characterization of γ‐Al_2_O_3_‐supported CuAu NP under CO oxidation by oxygen. a) Illustration of the combined in situ HE‐XRD/PDF and DRIFTS experimental setup.^[^ ^6^ ^]^ b) Scheme showing temperature and reaction gas changes for the in situ measurement (the catalyst was preheated at increments of 20 °C min^−1^ to 150 °C under helium (He) and kept for 10 min before exposure to CO + O_2_ at CO/O_2_ ratio of 1:5 for 1 h followed by cooling back to room temperature for 30 min under continuous CO + O_2_ atmosphere). c) A snapshot of the in situ HE‐XRD derived PDFs (Figure S17), comparing the experimental PDF and model computed PDF, for Cu_50_Au_50_/Al_2_O_3_ at 150 °C under He and CO + O_2_ reaction atmosphere (original atomic PDF: gray dots, model computed PDF: red curve; disordered fcc structure: blue curve; and ordered fcc structure: pink curve). d) Apparent “lattice parameters” estimated from model simulated PDFs (Figure S17) in terms of ordered fcc phase (bottom panel), disordered fcc phase (middle panel), and their ratio (top panel) versus time [(i) heating under He to 150 °C, (ii) CO + O_2_ reaction at 150 °C, and (iii) cooling down to room temperature]. e) A snapshot of time‐resolved in situ DRIFT spectra starting from stage ii (Figure S18), and spectral deconvolution showing CO_2_ and CO peaks. f) Plots of CO peak intensities versus time at stage ii (labeled as a and b of the time region) and stage iii (as indicated in the plots).

Having identified the atomic‐scale dynamic dual‐phase stability, the fundamental question is how it correlates with the “CP”. Since “CP” is an ensemble‐averaged surface activity of CO oxidation by O_2_ over the supported nanocatalyst, we hypothesized that there is a synergistic collaboration between the phase structure of the NPs and the surface‐active sites under the reaction condition. To prove the hypothesis, we tracked the ensemble‐averaged lattice and surface species under the catalytic reaction condition using combined in situ HE‐XRD/PDF and DRIFTS techniques (Figure 6a), which have recently demonstrated powerful applications in studying different nanoscale catalysts under various reaction conditions.^[^ ^6^, ^67^, ^68^, ^69^, ^70^ ^]^ The in situ HE‐XRD/PDF reveals the NPs’ phase structure, while the in situ DRIFTS entails the information on the surface‐active sites, both of which are obtained under the same reaction condition.

Using the combined in situ techniques, the dynamic phase structures and surface species/active sites of Cu_50_Au_50_/Al_2_O_3_ catalyst were measured under CO + O_2_ reaction condition (Figures 6 and S17,S18). The reaction temperature was set at 150 °C, which falls in the range of “CP” (Figure 1), by (i) heating under helium to 150 °C; (ii) reaction under CO + O_2_ for 1 h; (iii) cooling back to RT under CO + O_2_ (Figure 6b). Experimental PDFs from time‐resolved HE‐XRD data reveal a mixture of disordered (Fm‐3 m) and ordered (Pm‐3 m) fcc alloy phases (Figures 6c and S17) during the reaction process, showing evolution of the apparent “lattice parameters” in terms of Fm‐3 m, Pm‐3 m, and their volume ratios (Figure 6d). In stage (i), the disordered fcc phase exhibits moderate relaxation with a clear thermal expansion, while the ordered fcc phase remains almost unchanged, showing a disordered‐ordered volume ratio of about 68%. In stage (ii) at 150 °C, the alloy becomes more disordered and underwent a clear phase structure change. The “lattice parameter” remains at ∼3.510 Å for disordered fcc phase domains while showing a slight increase for the ordered fcc phase from 2.884 to 2.885 Å in the initial 1200 s. The volume ratio dropped by about 2%. After 1200 s, while the ordered phase significantly increases from 2.885 and 2.892 Å, the disordered phase shows little change. There is thus a significant drop in the volume ratio from 68% to 62%. Upon cooling down to RT (stage iii), the “lattice parameters” for the disordered and ordered phases remain at a relatively expanded state (3.511 Å for disordered and 2.890 Å for ordered phase), exhibiting an increased disordered‐to‐ordered volume ratio (73%∼79%). The results demonstrate that the relative ratio of disordered to ordered phase domains remains largely constant during CO oxidation at 150 °C despite subtle changes in disordered lattice parameters.

The evolution of the surface sites is assessed by simultaneously monitoring the surface reaction species under CO + O_2_ reaction condition using DRIFTS technique, including surface CO and CO_2_ species in the range of 2000–2400 cm^−1^ (Figures 6e and S18). The spectral peaks can be grouped into three regions based on FTIR and XPS studies,^[^ ^6^, ^42^, ^46^, ^70^, ^71^ ^]^ (Supporting Information): i') peaks at lower frequency (<2110 cm^−1^) assigned to Au^0^, and Au^δ‐^ or Au^0^ at edges or corners; ii') peaks at middle frequency (∼2140 cm^−1^) assigned to CO linearly adsorbed on Au^δ+^; and iii') peaks at higher frequency (>2170 cm^−1^) assigned to CO linearly adsorbed on Au^δ+^. In the lower frequency region (Figure 6e), the intensities of the two broad peaks at 2093 cm^−1^ (Au^δ−^) and 2112 cm^−1^ (Au^0^) are oscillatory (300 s period) at initial 1500 s (Figure 6f top). The three major peaks in the high‐frequency region, including 2191 cm^−1^ with a half‐height peak width of ∼ 9, 2181 cm^−1^ with a half‐height peak width of ∼8, and 2169 cm^−1^, are shown to follow similar patterns (Figure 6f bottom). The sharp peaks at 2191 and 2181 cm^−1^ are typically assigned to adsorption on a single atom,^[^ ^72^, ^73^ ^]^ reflecting non‐dipole‐dipole coupling interactions. Peaks in the middle‐frequency region (e.g., at 2138 and 2146 cm^−1^) were not observed in the initial ∼1500 s (Figure S19). Upon continuation of CO oxidation up to ∼3600 s, the CO adsorption peaks in low and high frequency regions mostly vanished. During the cooling down process, CO adsorption peaks in the low frequency region and at 2169 cm^−1^ appear to grow significantly as the temperature decreases, while the adsorption site assigned to single atom remains almost unchanged. The faster growth of intermediates in the low‐frequency region compared to the high‐frequency region indicates the less reactivity of CO adsorption species in the low‐frequency region. Interestingly, the gaseous CO bands at 2172 and 2115 cm^−1^ appeared every 300–400 s in stage (ii) at initial ∼1700 s followed by complete disappearance up to ∼3600 s. The bands at ∼2190, ∼2112, and ∼2057 cm^−1^ are assigned to CO species linearly adsorbed on at edges or corners. The surface sites feature a combination of Au^δ+^ and Au^0^ species with the Cu_2_O as revealed previously by XPS characterization.^[^ ^41^, ^71^ ^]^ The in situ DRIFTS monitoring of the surface species revealed oscillatory peaks, with the low‐frequency peaks (2093–2112 cm^−1^) corresponding to atop CO on Au^δ−^ site, and the high‐frequency peaks (2169–2191 cm^−1^) corresponding to atop CO on Au^δ+^ site^[^ ^38^ ^]^ (Figure 6f).

The surface species seems to exhibit an oscillatory characteristic in terms of “appearance and disappearance” of the peaks associated with gaseous CO_2_ (∼2360 cm^−1^) and linearly adsorbed CO_2_ on Cu (2340 cm^−1^) every 300–400 s in the initial 1700 s. During cooling down to RT, CO_2_ peaks disappear, while carbonate species (1650 cm^−1^) and linearly adsorbed CO on Al_2_O_3_ (1690 cm^−1^) evolve, showing significant growth of CO on Au^0^ (∼2112 cm^−1^) with weak growth of CO on Au^δ+^ and Au^δ‐^ sites (Figure 6f). Data from the time dependence of the peak intensity increase at 2112 cm^−1^ reveal that CO linearly adsorbed on Au^0^ site is responsible for the catalytic activity drop as temperature decreases. The irregular oscillation patterns shown in the DRIFTS spectra (see Figure 6) can be further assessed by Fast‐Fourier transform (FFT) and Hilbert–Huang transform (HHT) analysis (see Supporting Information and Figure S20a‐v), which would allow simulation of individual regular oscillation frequencies.^[^ ^74^ ^]^ A combination of the individual regular frequencies leads to irregular oscillation, which reflects the differences in size distribution and surface sites. While the phase and lattice parameter remains almost unchanged, except for a relaxation of fcc disordering with lattice expansion, the oscillatory pattern reveals dynamic changes in the adsorption and desorption of the surface species. The oscillatory feature is indicative of two or more possible surface‐active sites for the adsorption and desorption of CO or CO_2_ on Au^δ+^ (with O‐species on Cu atoms) and Au^δ‐^ sites (without O‐species on Cu atoms) during the surface reactions (Figure S20w). For CO oxidation over supported transition metal catalysts, possible active oxygen species from O_2_ include O^2−•^, O_2_^2−^, O^−•^, and O^2−^ (adsorbed and lattice oxygen).^[^ ^75^ ^]^ The surface sites are linked to the formation of activated oxygen species and refilling of oxygen vacancies,^[^ ^76^ ^]^ in which synchronization could lead to oscillatory kinetics. The “regular‐irregular” oscillations for the CO_ads_ peaks occur mainly at the initial stage of the reaction and the temperature cooling‐down stage. In addition, the narrow peak width (FWHM < 10 cm^−1^) for some of the peaks is indicative of CO atop adsorption on a single‐atom site.^[^ ^77^, ^78^ ^]^ The implied high mobilities of atoms on the support surface during the reaction is yet to be confirmed by higher resolution in situ TEM, along with composition mapping. Nevertheless, the NP size distribution could lead to differences in atomic sites and mobilities on the surface/subsurface in several possible steps, including O_2_ dissociative adsorption in competitive co‐adsorption, formation of surface/subsurface O leading to Cu_2_O phase segregation with surface Au‐enrichment, an increase in CO adsorption rate by reaction with activated O, a decrease in reaction rate due to O depletion, and O_2_ dissociative adsorption to refill O for the next cycle. Such structural oscillation is consistent with lattice parameter changes under O_2_ and H_2_ (see Figure 5), and cyclic deoxygenation of CuO surface during O_2_ + H_2_ reaction, which is accompanied by segregation/desegregation of Au atoms as revealed in our previous work.^[^ ^41^ ^]^

Overall, the disordered fcc lattice remains constant despite the lattice expansion in the ordered phase, and there is an oscillatory CO adsorption which diminishes under prolonged CO oxidation conditions. The alloy structure exhibits a clear decrease in the disordered‐to‐ordered volume ratio and lattice expansion. This dynamic surface oxidation state and the associated redistribution of charge over the alloy atoms in terms of Au^0^ and Au^δ+^ species are responsible for the sustainable activity. The findings are further supported by in situ AP‐XPS characterization of AuCu NPs of different compositions,^[^ ^41^, ^71^ ^]^ and surface Cu promotes dissociative O_2_ adsorption to produce atomic O species. It is the enrichment of Au in the subsurface region that hinders the inward incorporation of atomic O into the NP.^[^ ^41^ ^]^ Under catalytic CO + O_2_ condition, while Au‐rich NPs (Cu_75_Au_25_) fully oxidize into a complete CuO_x_ shell, Au‐poor (Cu‐rich) NPs (Cu_90_Au_10_) can retain stable Au clusters within the CuO_x_ shell.

The dynamic stability is further assessed in terms of the NP‐NP and NP‐support interactions. Preliminary molecular dynamics (MD) simulations of CuAu NPs under elevated temperatures (Figure S21) suggest that the interparticle necking of un‐supported alloy NPs is dominant, leading to transient changes in potential energy curves (Figure S21a–e). For a preliminary model of the alloy NP on Al_2_O_3_ under oxygen, the simulation result shows that the NP height decreases, while the contact area increases with temperature (Figure S21f–m). This result reflects a strong temperature dependence of the CuAu alloy NP‐Al_2_O_3_ interaction (adhesion). The trend for the relatively stabilized NP height and contact area at ∼150 °C may suggest a “buffering effect” of the NP‐support interaction on the structure and morphonology responsible for the dynamic stability, which seems to be consistent with the trend shown by the cross‐NP line profiles shown earlier (Figure S8). However, this result is preliminary, but points to potential correlation for the temperature dependence in further in‐depth simulations.

## Conclusion

In summary, the results from in situ ETEM tracking of alumina‐supported ultrasmall CuAu NPs under oxygen at different temperatures have revealed dynamic atomic‐scale and nanoscale crystalline and amorphous phase structures. The dynamic stability of the atomic‐scale phase structures over an elevated temperature range (100°C∼200 °C) coincides with the catalytic conversion plateau observed in the temperature dependence of carbon monoxide oxidation on the catalyst. Aided by combined operando HE‐XRD/DRIFTS characterizations under CO oxidation, the understanding of the unique morphological‐catalytic correlation is further substantiated by the dynamic ordering/disordering phase structures and irregularly oscillatory surface species under carbon monoxide oxidation. This finding represents the first example demonstrating the atomic‐scale dynamic phase structures in correlation with the ensemble‐average dynamic ordering/disordering phase structures and surface sites. The insights into the unique catalytic synergy of the alloy NPs under reaction conditions have implications for the design of active and stable ultrasmall alloy catalysts at elevated reaction temperatures and for further in‐depth experimental and computational studies of deactivation‐resistant alloy catalysts in terms of size, composition, phase, and surface chemistry.

## Supporting Information

The authors have cited additional references within the Supporting Information.^[79–88]^

## Conflict of Interests

The authors declare no conflict of interest.

## Supporting information



Supporting Information

## Data Availability

The data that support the findings of this study are available in the supplementary material of this article.
