# Supplementary material for: In Situ/Operando Probing of Dynamic Phase Structures of Alumina‐Supported Ultrasmall Copper‐Gold Alloy Nanoparticles Under Reaction Conditions
Source: Angew Chem Int Ed Engl. 2025 Jun 16;64(32):e202508735. doi: 10.1002/anie.202508735 (PMC12322633; doi:10.1002/anie.202508735)
Supplement: Supplementary file 1 — Supporting Information [file ANIE-64-e202508735-s001.docx]

Supporting Information

In-Situ/Operando Probing of Dynamic Phase Structures of Alumina-Supported Ultrasmall Copper-Gold Alloy Nanoparticles under Reaction Conditions

Han-Wen Cheng^[a],[b]^, Jing Li^[b]^, Shiyao Shan^[b]^, Xiaowei Lv^[a]^, Guanyu Chen^[a]^, Merry Madiou^[b]^, Dong Dinh^[b]^, Seyed-Danial Mousavi^[b]^, Zhipeng Wu^[b]^, Shan Wang^[b]^, Yazan Maswadeh^[c]^, Valeri Petkov^[c]^, Susan Lu^[b]^, Ke Pei^[a]^, Wenbin You^[a]^, Renchao Che*^[a]^, and Chuan-Jian Zhong*^[b]^

Table of Contents

Materials & Methods………………………………………………………...S2-S5

Supporting Figures……………………………………………………….....S6-S16

References………………………………………………………………………S17

**Materials & Methods**

***Chemicals***. Tetrachlorohydrogenaurate trihydrate (HAuCl_4_·3H_2_O, 99%), copper (II) chloride dihydrate (CuCl_2_·2H_2_O 99%), 1-dodecanethiol (CH_3_(CH_2_)_11_SH, 98%), sodium acrylate (H_2_C=CHCO_2_Na), and 4-Nitrophenol (4-NP) were purchased from Sigma-Aldrich. Aluminum oxide pellet (43832) was purchased from Alfa Aesar, titanium(IV) oxide (Aeroxide™ P25) powder were purchased from Fisher Scientific, Vulcan carbon XC-72 was obtained from Cabot. Other chemicals including sodium hydroxide (NaOH), sodium choloride (NaCl), and hexane were purchased from Fisher Scientific. Gases of 1 vol% CO balanced by N_2_ and 20 vol % O_2_ balanced by N_2_ were obtained from Airgas. All chemicals were used as received. Water was purified with a Millipore Milli-Q water system (18.2 M Ohm).

***Synthesis of bimetallic CuAu nanoparticles and catalyst preparation****.* The CuAu NPs was synthesized by a modified seeded growth method based on our previous work.^[79-82]^ Typically, the Au and Cu precursors with different ratios concentrations were mixed under vigorous stirring in 100 mL milliQ water followed by adjusting pH to neutral with 1.0 M NaOH. Sodium acrylate was used as both reducing reagent and capping reagent. The solution was kept stirred under N_2_ gas or under air at room temperature. The resulting purple products were collected and stored under ambient atmosphere. The bimetallic composition was controlled by the feeding ratio.

The as-synthesized NP prepared under N_2_ atmosphere, e.g., Cu_50_Au_50_ NPs, features a five-fold multiply twinned structure, which is supported by analysis of fast Fourier transform pattern. The structure exhibits a lower total energy due to abundance of {111} surfaces at the expense of internal strains.^[83]^ The interplanar spacing 0.212 nm is characteristic of {111} plane. In contrast to the tendency of aggregation of the NPs in aqueous media due to weak surface capping (3.9 ±0.7 nm) (Figure S2a), highly monodispersed distribution is evident after phase transfer of the NPs using DDT into hexane media (3.4 ±0.4 nm) (Figure S2b). The analysis of EELS mapping revealed uniform distribution across the NPs. The NPs feature mainly face-centered cubic (fcc) based on XRD patterns (Figure S2c). The fcc-lattice parameter for the NPs shows a positive deviation from the linear dependence predicted by Vegard’s law, indicating that the nanoalloys are under substantial tensile stress. Cu_50_Au_50_ showed the most deviation.

The formation of the bimetallic NPs was also followed by monitoring the surface plasmonic resonance band using UV-Vis spectroscopic method as a function reaction time. At the initial 24 – 48 hrs, the plasmonic band of Cu_50_Au_50_ reveals a broad peak at the range of 500 nm to 800 nm, indicating the formation of ultrasmall CuAu nanoparticles.^[84]^ The longer time leads to a blue shift at 518 nm with narrower band width.

Cu*_n_*Au_100-_*_n_* nanoparticles were synthesized under ambient atmosphere followed by assembly on different supports and then treated under O_2_ at 260 ^o^C for 1 hr before catalytic evaluation. To prepare supported catalysts, the Cu*_n_*Au_100-_*_n_* nanoparticles were cleaned by centrifugation by 50% ethanol solution for three times and redispersed in DI water. Then controlled amounts of Cu*_n_*Au_100-_*_n_* NPs were mixed with γ-alumina (Cu*_n_*Au_100-_*_n_*/Al_2_O_3_) or carbon (Cu*_n_*Cu_100-_*_n_*/C) under sonication and stirred overnight respectively. The resulting supported catalysts were dried under N_2_ atmosphere and collected for activation before testing. Typically, the supported catalysts were activated by thermochemically treating under 20 vol % O_2_ for 1 h at 260 °C to remove capping reagent on the nanoparticles. In this work, the weight loadings for Cu*_n_*Au_100-_*_n_* catalysts were around 1%. The exact mass loading was determined by Inductively Coupled Plasma Mass Spectrometer (ICP-MS).

***Characterization and Instrumentation***. *UV-Vis.* The UV-Visible absorption spectra were collected by HP 8453 spectrophotometer equipped with Deuterium lamp and Halogen lamp as light source in the range of 200-1100 nm. Absorption spectra of Cu*_n_*Au_100-_*_n_* nanoparticle were collected at regular time intervals. The samples were pre-diluted by deionized water for UV-Vis measurement.

*Environmental transmission electron microscope (ETEM).* The in-situ experiments were carried out using Thermo Fisher Themis Z ETEM (Themis ETEM D3669). There is an attachment with an in-situ gas supply system (300 KeV). A differential pump system was used to maintain the pressure difference between the sample region (1 Pa) and the TEM column (2×10^-5^ Pa). An optimal spatial resolution of 70 pm was achieved. A heating holder was used to control the temperature. The heating rate was set at 1 °C/second. A MEMS microheater was used for heating. The sample was loaded on a chip made of amorphous silicon carbide.

The software used for FFT analysis was Velox (Thermos Fisher Scientific Inc.). Briefly, in the display settings side panel, select Fast Fourier Transform (FFT), then the object properties panel displays the name, image size, pixel size and windowing mode (Hann, Hamming, BlackmanHarris). Before performing FFT on a signal, it is usually necessary to multiply the signal by an operational function. This is done to reduce spectral leakage and frequency aliasing that occur due to signal truncation. Different functions, such as the cosine, Hamming, Hanning, and Gaussian functions, produce different effects in the frequency domain. The choice of the function depends on the characteristics of the signal and analysis requirements. In this work, the Hann function was chosen.^[85]^

*Transmission Electron Microscopy (TEM)*. The morphology of Cu*_n_*Au_100-_*_n_* nanoparticles and supported Cu*_n_*Au_100-_*_n_* catalysts were measured by JEM 2100F from JEO. The high-resolution transmission electron microscopy (HRTEM) images were obtained by using aberration-corrected ACAT microscopy at Argonne National Laboratory (ANL). The composition distribution of Cu*_n_*Au_100-_*_n_* nanoparticles was determined by high-angle annular dark-field scanning TEM (HAADF-STEM) equipped with energy dispersive X-ray spectrometer (EDS) from FEI Talos F200X at Argonne National Laboratory (ANL) and Brookhaven National Laboratory (BNL). The samples were prepared by dropping cast of Cu*_n_*Au_100-_*_n_* nanoparticles or supported Cu*_n_*Au_100-_*_n_* nanoparticle catalysts onto an ultrathin carbon film on lacey carbon support film and lacey carbon type-A respectively followed by solvent evaporation at room temperature. The size distributions of Cu*_n_*Au_100-_*_n_* nanoparticles were statistically calculated by using the ImageJ software.

*X-ray Diffraction (XRD).* XRD was used to identify the lattice constant and estimate particle size of Cu*_n_*Au_100-_*_n_* NPs. The powder XRD patterns were recorded on a Phillips X’pert PW 3040 MPD diffractometer with Cu Kα source (λ = 1.5418 Å), equipped with a sealed Xe proportional detector. The data was collected from 2*θ* = 10^o^ to 90^o^ at a scan rate of 0.04 per step and 0.04 degree per second*.* The XRD patterns were compared with the International Centre for Diffraction Data (ICDD) database for phase analysis.

*X-ray photoelectron spectroscopy (XPS).* XPS measurements were performed on a Physical Electronics Quantum 5000 scanning ESCA microprobe with a focused monochromatic Al Kα X-ray (1486.7 eV) source for excitation. The X-ray beam size was 100-μm in diameter and the estimated spot size is 1.4 mm × 0.2 mm on sample. This system equipped with 16 element multichannel detector and a spherical section analyzer. The binding energy (BE) was calibrated using a C 1s peak at 284.8 eV as an internal standard.

*Inductively Coupled Plasma Mass Spectrometer (ICP-MS)*. The composition of Cu*_n_*Au_100-_*_n_* NPs and the loading of supported Cu*_n_*Au_100-_*_n_* catalyst was determined using a Perkin Elmer Elan 6000 ICP-MS instrument equipped with a simultaneous extended dynamic range detector and a cross-flow nebulizer. The parameters are plasma 18.0 L Ar(g)/min, auxiliary 0.3 L Ar(g)/min, nebulizer 0.73 L Ar(g)/min, power 1500 W, and peristaltic pump rate 1.40 mL/min. A Meinhardt nebulizer coupled to a cyclonic spray chamber was used to increase sensitivity with the parameters of 18.0 L Ar(g)/min, auxiliary 0.3 L Ar(g)/min, nebulizer 0.63 L Ar(g)/min, power 1500 W, peristaltic pump rate 1.00 mL/min. The standards were analyzed for every 6 samples to check, with instrument re-calibration if check standards were not within 5% of the initial concentration.

*Combined In-situ Diffuse reflectance infrared Fourier transform spectroscopy (DRIFTs)* *and High-Energy X-ray Diffraction (HEXRD).* The combined DRIFTS and HEXRD measurements were carried out using a Bruker Vertex 70 FTIR spectrometer with an MCT detector and a modified Praying MantisTM Diffuse Reflectance Accessory (Harrick Scientific Products, Inc.) at Sector 11-ID-B at ANL. Briefly, the sample cup in Praying MantisTM Diffuse Reflectance Accessory was filled up with around 30 mg for Al_2_O_3_ supported catalysts and then heated up to reaction temperature (e.g. 150 ^o^C) under 20 mL/min helium for 10 mins for background collection before exposure to a mixture of carbon monoxide and oxygen balanced by helium atmosphere at a constant total flow rate of 20 mL/min. Each DRIFTs spectrum was collected with a nominal resolution of 2 cm^-1^ and 128 scans for every 100 second. The high Energy X-ray Diffraction Data were collected every 4 – 5 mins using X-rays with a wavelength, λ, of 0.1080 Å (X-ray energy ∼115 keV). The scattered intensities were collected with a large area (amorphous Si) detector. The cell was mounted on a Bruker Vertex 80 spectrometer equipped with a Praying mantis-type optics. The optics allows DRIFTS measurements from a horizontal sample surface while the surface is probed with an x-ray beam. The ability to control the vertical size and position of the incoming x-ray beam ensured that the infrared (IR) and x-ray beams probed coinciding sample volumes.

Experimental XRD data were corrected for experimental artifacts, reduced to the so-called structure factors, S(*q*), and then Fourier transformed to atomic PDFs G(*r*) using a wave vector defined as *q*=4πsin(*θ*)/*λ*, where *θ* is half of the scattering (Bragg) angle and *λ* is the wavelength of x-rays used. Note, as derived, atomic PDFs G(*r*) are experimental quantities that oscillate around zero and show positive peaks at real space distances, *r*, where the local atomic density *ρ*(*r*) exceeds the average one *ρ_o_*. Details of the in-situ HE-XRD/PDF and DRIFTs techniques for the study of catalysts under gas phase reaction conditions are described previously.^[6]^

***Catalytic performance testing.*** The in-house catalytic activity measurement was conducted using a customer-built testing station with a continuous flow of carbon monoxide (CO, 1 vol.% balanced by He) + O_2_ (20 vol.% balanced by N_2_) at 20 mL/min through the fixed catalyst bed in quartz reactor including a temperature-controller, gas flow/mixing/injection controllers, and an on-line gas chromatograph (Shimadzu GC 8A) equipped with 5A molecular sieve and Porapak Q packed columns and a thermal conductivity detector (TCD). The catalysts were loaded in a quartz micro-reactor tube (inner diameter: 4 mm) and wrapped by quartz wool in the middle of the tube (length of the catalyst bed: 6 mm). The residence time was about 0.09 seconds. Gas hourly space velocity (GHSV) used in this work is approximately 40,000 h^-1^. The conversion of CO oxidation reaction was determined by analyzing the composition of the tail gas effusing from the quartz micro reactor packed with catalyst fixed bed.

***Computational Modeling*.** DFT calculations were carried out using DMol^3^ package in the Materials Studio Software. The Perdew–Burke–Ernzerhof (PBE) functional with a generalized gradient approximation (GGA) was used to describe the exchange-correlation interaction. The spin unrestricted DFT calculations were performed and a double-numerical basis set with polarization functions (DNP) was used. The fcc plane structure was used to study the CO adsorption energy on the surface. A 15 Å vacuum along the z-direction was used between the repeated slabs. The adsorption species described here were fully relaxed and ∆*E*_ads_ = *E*_CO/CuAu_  - *E*_CuAu_ was used to calculate the adsorption energies of CO on the surface, where *E*_CO/CuAu_, and *E*_CuAu_ are the total energy for the CO adsorbed CuAu system and CuAu system, respectively.

***Time-Frequency Data Transformations.*** The oscillation data were analyzed by Fast-Fourier Transform (FFT) and Hilbert–Huang Transform (HHT). FFT assesses oscillation frequencies by analyzing stationary data, whereas HHT is a statistical/empirical approach to oscillation frequencies by analyzing non-stationary and non-linear data. In FFT, an oscillation signal is converted from its original time domain to a representation in the frequency domain and vice versa. Discrete Fourier transform was obtained by decomposing a sequence of oscillatory patterns into components of different FFT frequencies. FFT refers to a way the discrete Fourier Transform. The HHT method is a fully data-driven and adaptive method for analyzing non-stationary and nonlinear signals.^[86]^ HHT uses Empirical Mode Decomposition (EMD)^[74]^ to process oscillation data in our analysis. HHT’s unique advantage towards more accurate and quantitative analysis is originated from its superior capability to reveal both global information (i.e., EMD enabled IMFs) and local information (Hilbert spectral analysis) from any input signal. The EMD method is a decomposition process. The decomposition assumes that any data contains different simple intrinsic modes of oscillations, and each decomposed mode is known as an IMF.^[74]^

**Figures:**


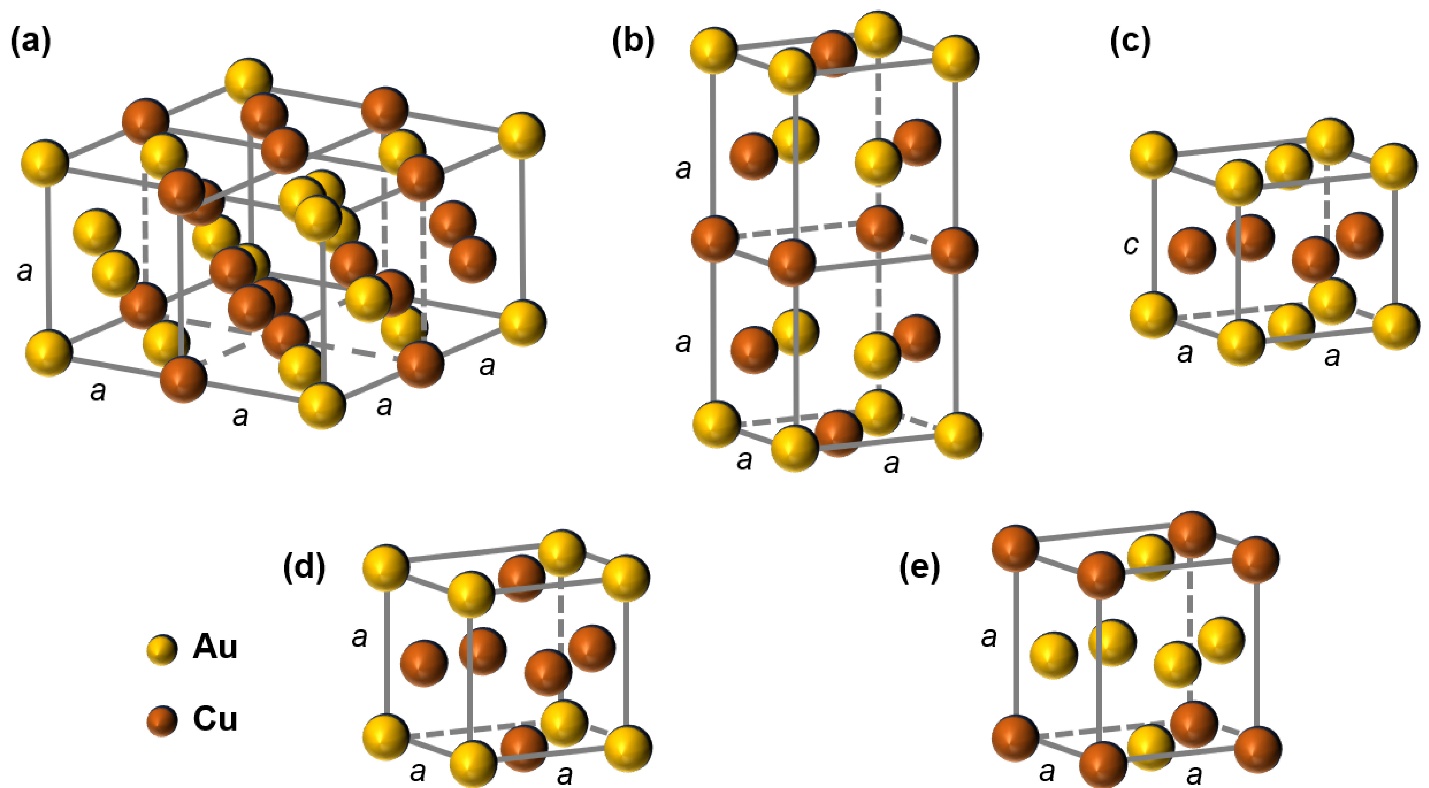


**Figure S1.** Illustration of unit cell structures of fcc (CuAu) (a, 4 × 1 supercell, *a*=3.89 Å; b, 1 × 2 supercell, *a*=3.89 Å), L1_0_ fct (CuAu) (c, 1 × 1 cell, *a*=3.89 Å, and *c*=3.67 Å), and L1_2_ order-fcc (Cu_3_Au (d, *a*=3.74 Å) and Au_3_Cu (e, *a*=4.088 Å), space group: Pm-3m).


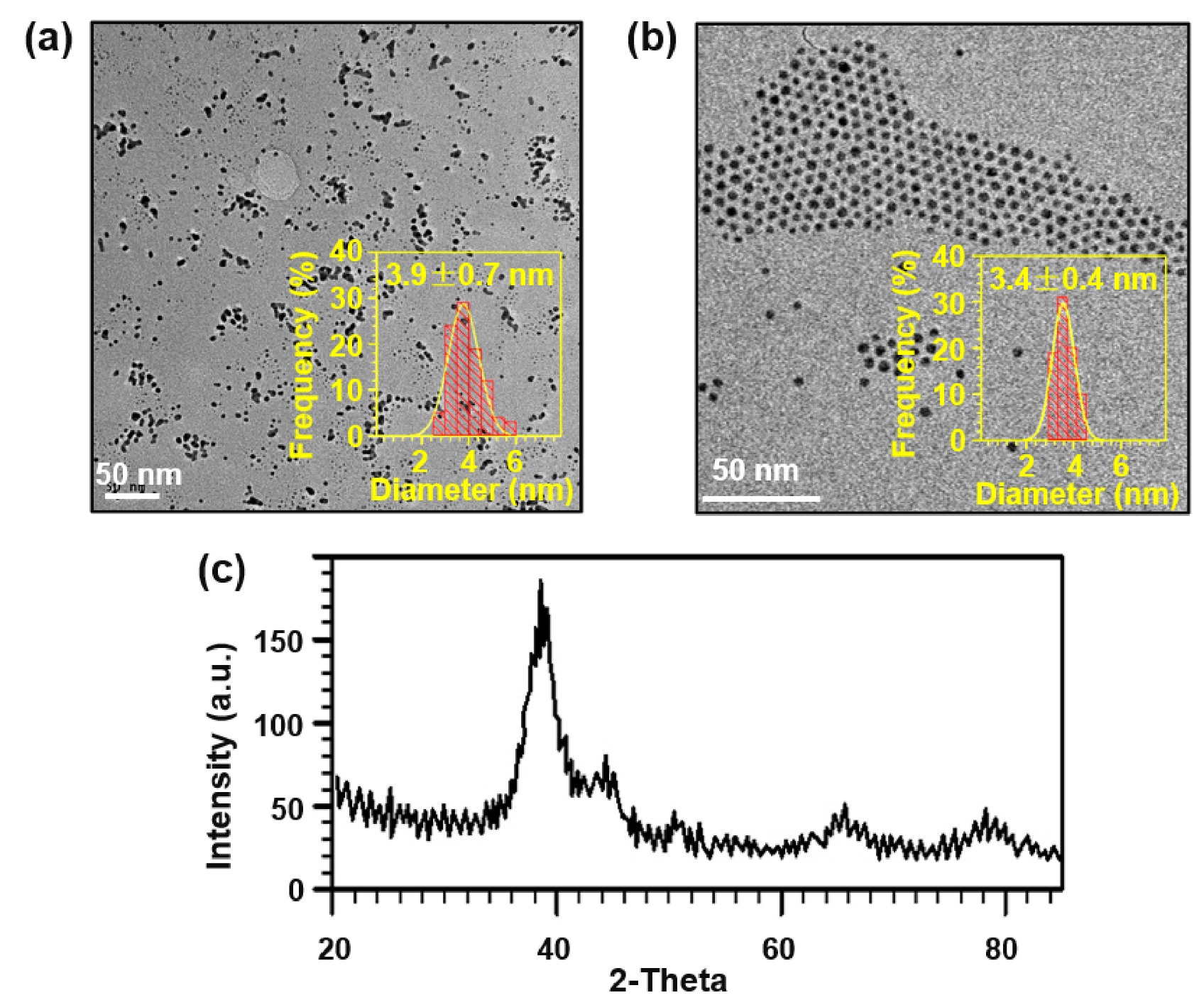


**Figure S2.** (a-b) A representative set of TEM images of Cu_50_Au_50_ nanoparticles: (a) As-synthesized in the aqueous solution (3.9 ±0.7 nm); and (b) after being transferred to hexane solution (3.4 ±0.4 nm). (c) X-ray diffraction pattern of Al_2_O_3_-supported Cu_50_Au_50_ NPs.


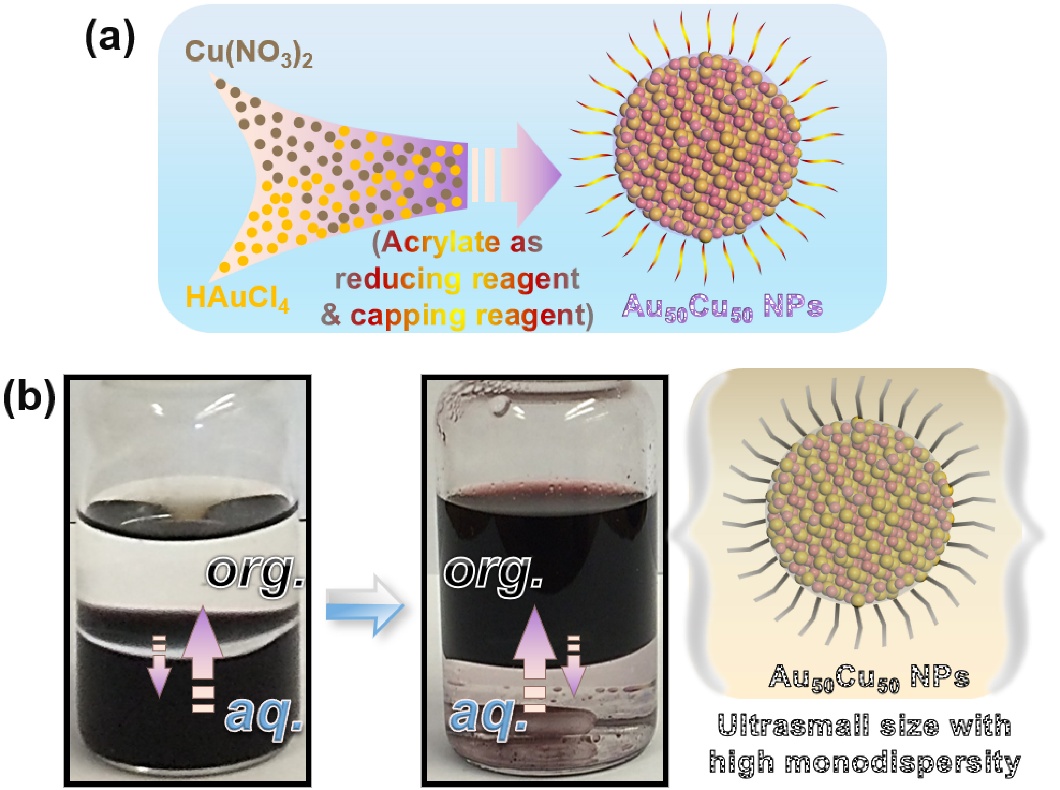


**Figure S3**. Synthesis and processing of CuAu alloy nanoparticles. (a) Scheme showing bottom-up reduction of Au and Cu precursors by acrylates (CH_2_CHCO_2_^-^) to form CuAu NPs in aqueous solution. (b) Photos showing phase transfer to organic phase ligand exchange reaction (DDT, 1-dodecanethiol, CH_3_-(CH_2_)_12_-SH) in organic solution to replace acrylates on the NP. Note that atoms, particles, and capping molecules (CA) are not drawn to scale.


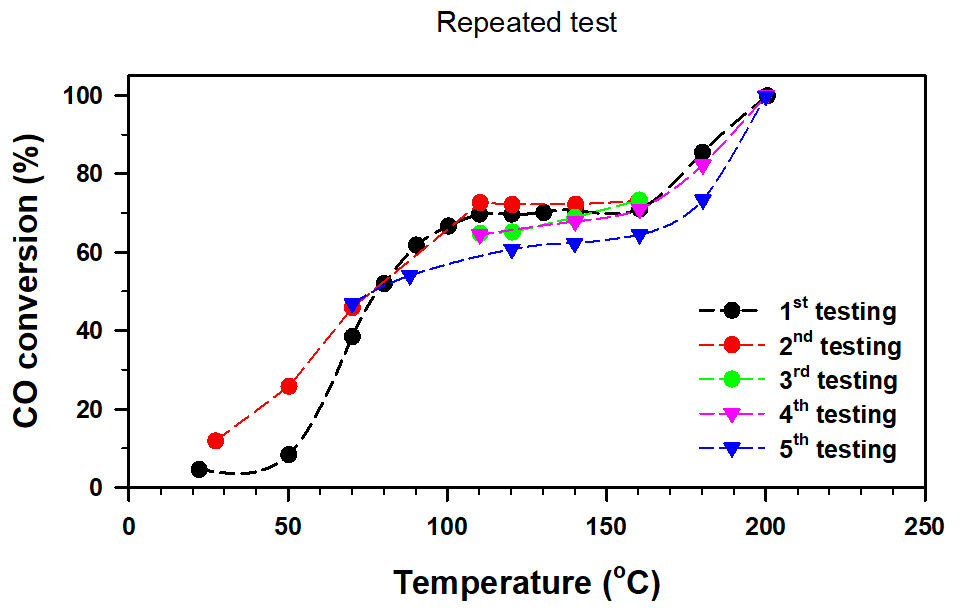


**Figure S4.** Multiple replicates of the catalytic carbon monoxide oxidation activity of the catalyst Cu_50_Au_50_/Al_2_O_3_.


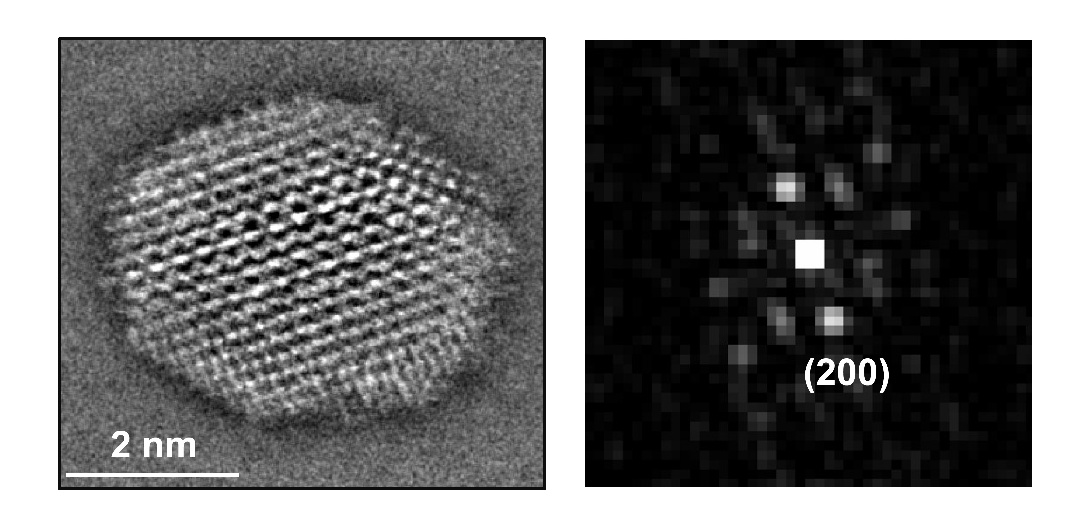


**Figure S5.** TEM image and FFT (Fast Fourier transform) pattern for the γ-Al_2_O_3_-supported CuAu NP (3.9-nm) in vacuum.


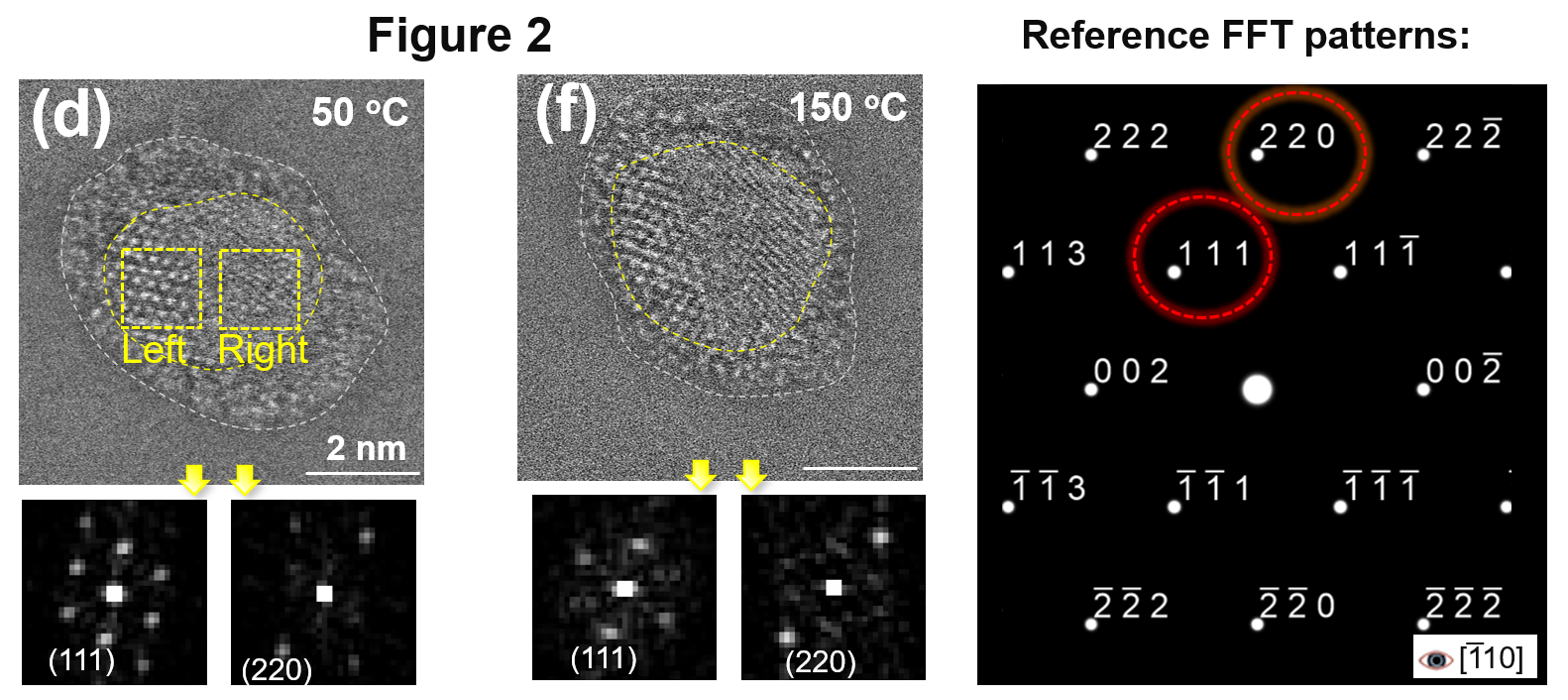


|  | *d*-spacing (in nm) based on FFTs | | | |
| --- | --- | --- | --- | --- |
|  | left box | assigned: | right box | assigned: |
| Figure 2d | *d*(111)=  0.225 | CuAu  (fct) | *d*(220)=  0.136 | Cu_3_Au |
| Figure 2f | *d*(111)=  0.228 | CuAu  (fct) | *d*(220)=  0.152 | Cu_2_O |

| Reference FFT pattern (*d*-spacing in nm) | | | | |
| --- | --- | --- | --- | --- |
| Cu_2_O | Au_3_Cu | Au | CuAu (fct) | Cu_3_Au |
| *d*(111)=  0.246 | *d*(111)=  0.236 | *d*(111)=  0.235 | *d*(111)=  0.223 | *d*(111)=  0.216 |
| *d*(220)=  0.153 | *d*(220)=  0.147 | *d*(220)=  0.147 | *d*(220)=  0.138 | *d*(220)=  0.135 |

**Figure S6.** Top panel: reference FFT patterns. Bottom panel: two tables showing examples of the *d*-spacing values calculated based on the FFT patterns in Figure 2d and f and the reference FFT patterns. These *d*-spacing values are for the two domains of *c-* phase regions in terms of (111) and (220) planes of CuAu (fct), Cu_3_Au (Pm-3m) and Cu_2_O in the [-110] direction.^[87,88]^


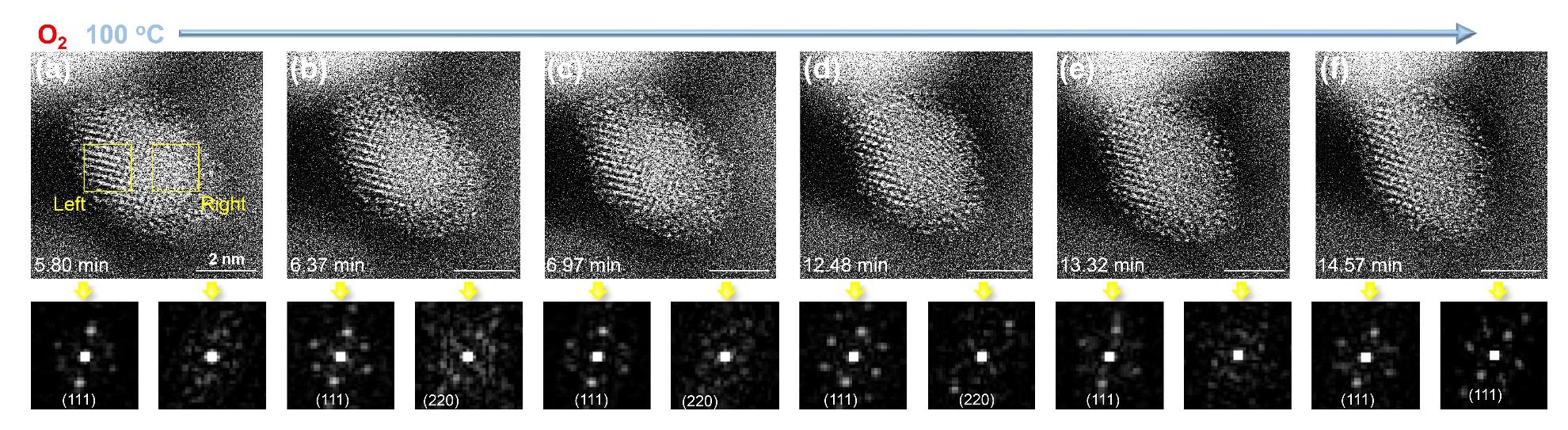


**Figure S7**. Isothermal in-situ TEM images of γ-Al_2_O_3_-supported CuAu NP (with an initial *c-*size: 3.9-nm, the same NP as in Figures 2 and 3) under O_2_ at 100 ^o^C. Bottom panel: FFT patterns (as illustrated by left and right dash-box in image). Scale bars: 2.0 nm.


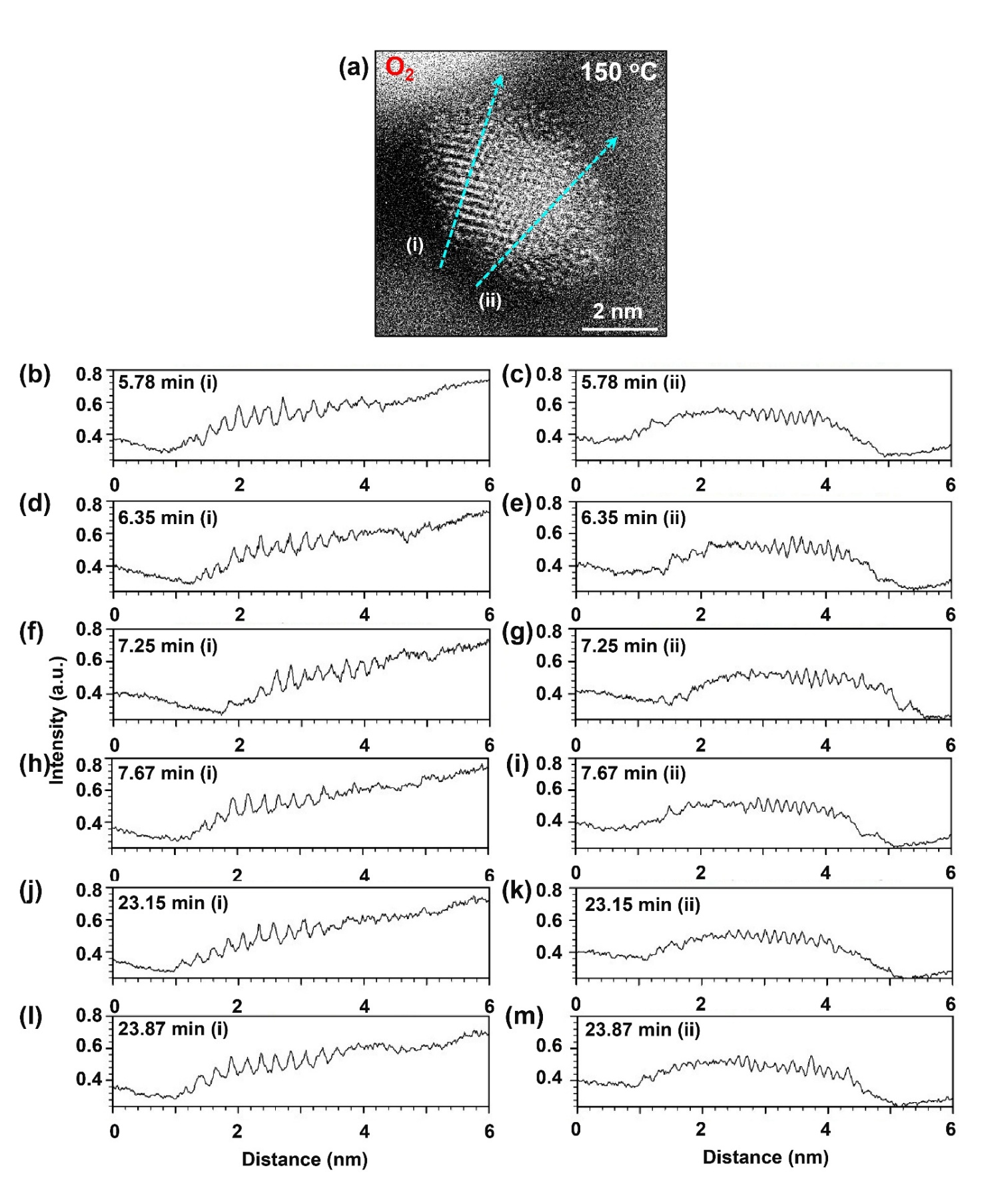


**Figure S8.** (a) In-situ TEM images of the 3.9 nm NP (corresponding to the NP shown in Figure 3) under O_2_ (20Pa) and line intensity profiles (b-m) as illustrated by the cross lines in image-a.


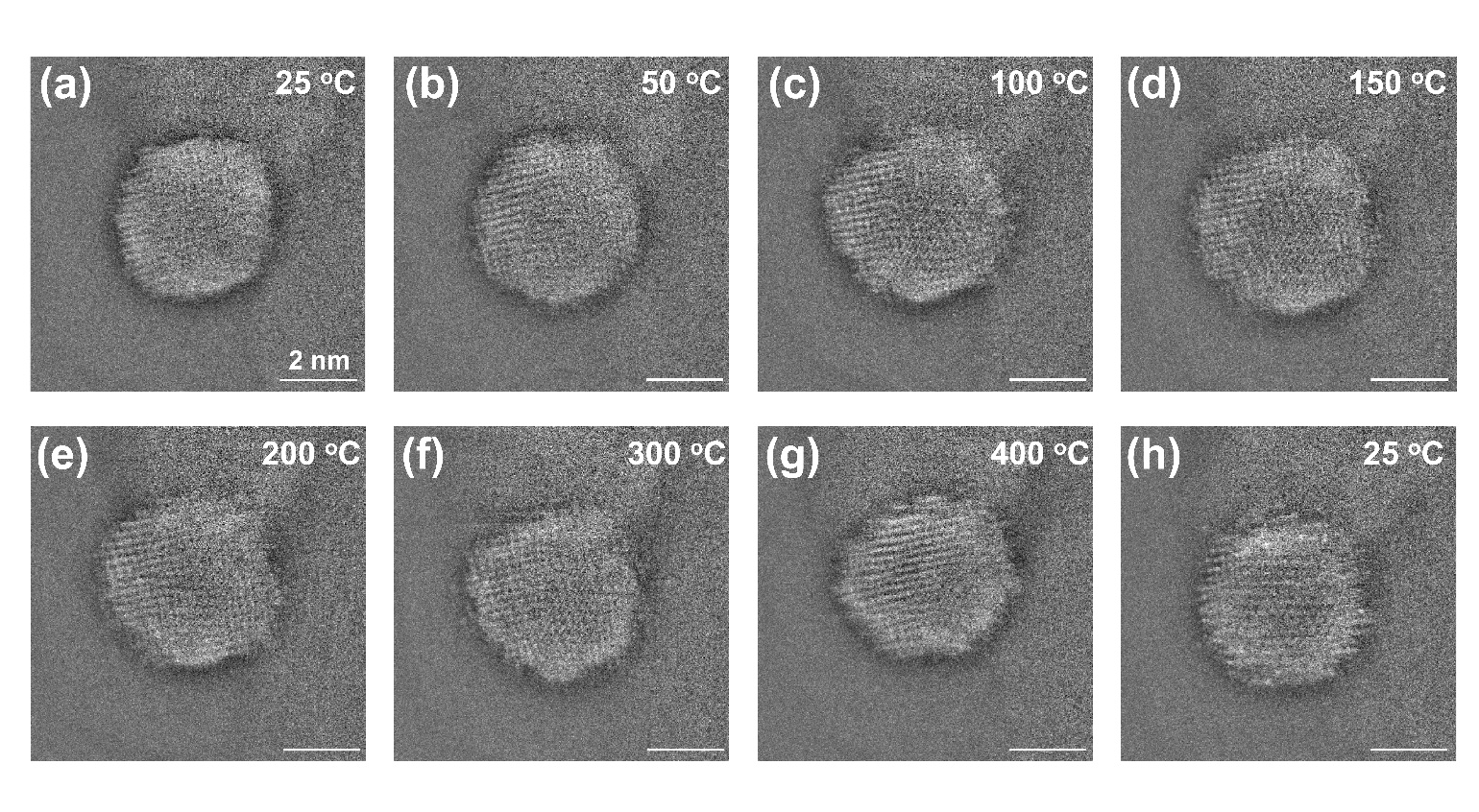


**Figure S9**. In-situ ETEM tracking of the temperature dependence of the atomic phases of γ-Al_2_O_3_-supported CuAu NP (3.9-nm) under H_2_ (20 Pa). Changing the temperature range from 25 (a) to 400 ^o^C (g) and back to 25 ^o^C (h). Scale bars: 2.0 nm.


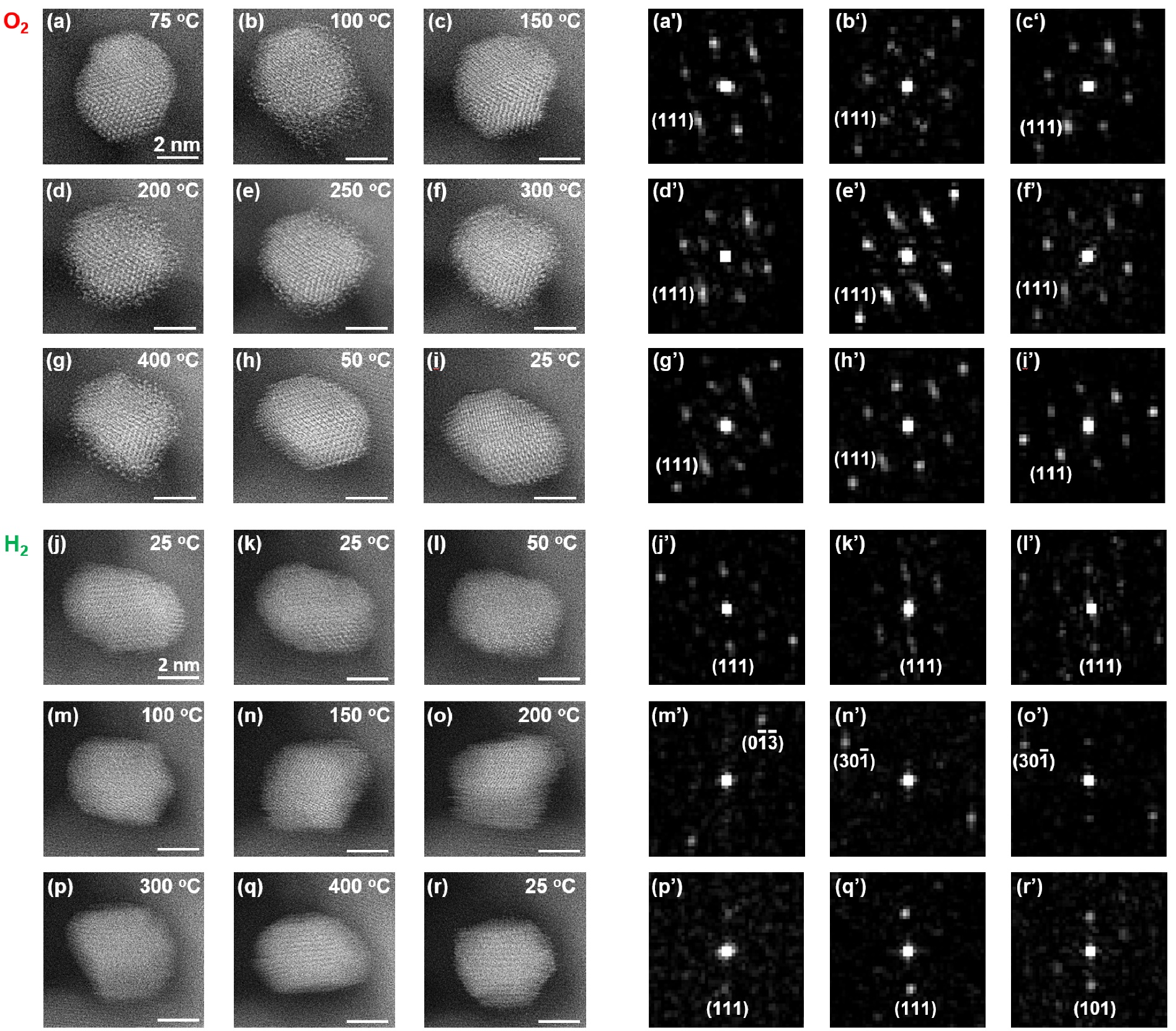


Figure S10. In-situ ETEM tracking of the temperature dependence of the atomic phases of γ-Al_2_O_3_-supported CuAu NP (5-nm) under O_2_ (a-i) and H_2_ (j-r). Right: corresponding FFT patterns.


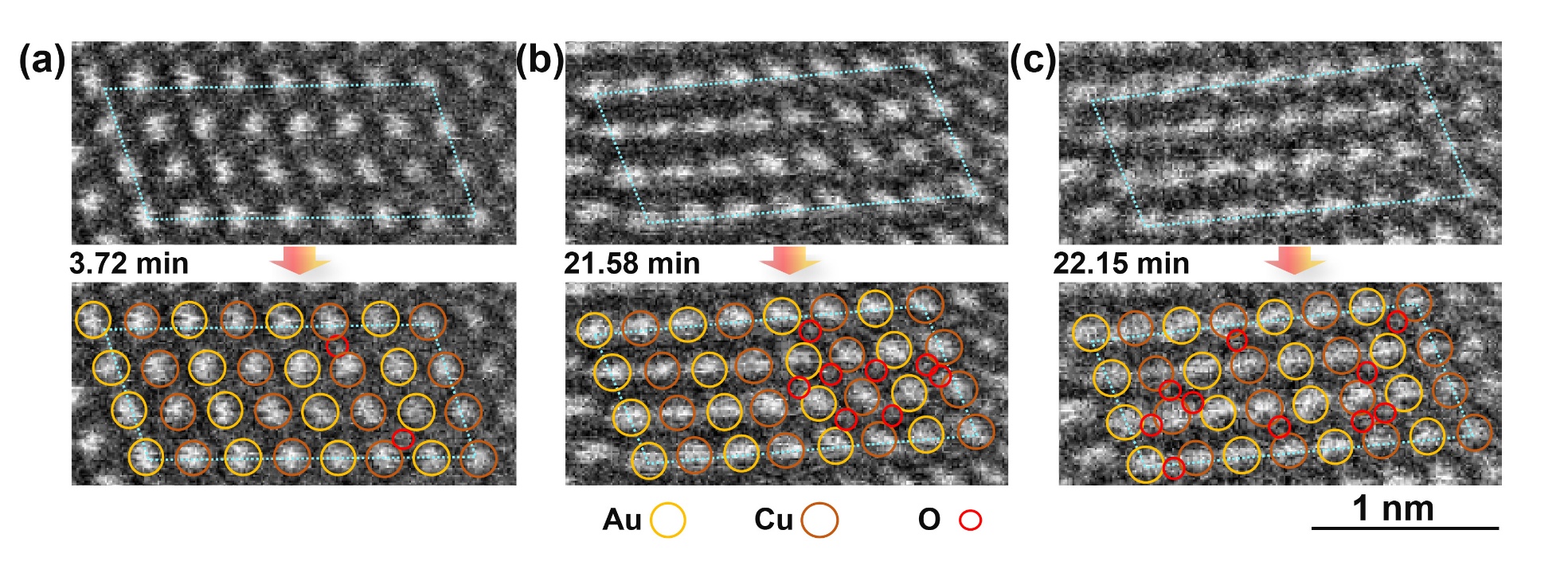


**Figure S11.** Magnified in-situ TEM image of 6.5-nm NP (high-pass mode, same NP as in Figure 4) at 3.72 (a), 21.58 (b), and 22.15 (c) min. The corresponding dynamic atomic mobility/diffusion of the metal atom/vacancy, lattice oxygen/vacancy or interstitial oxygen (O_L/V/I_) is illustrated by the color circles. Scale bar: 1 nm.


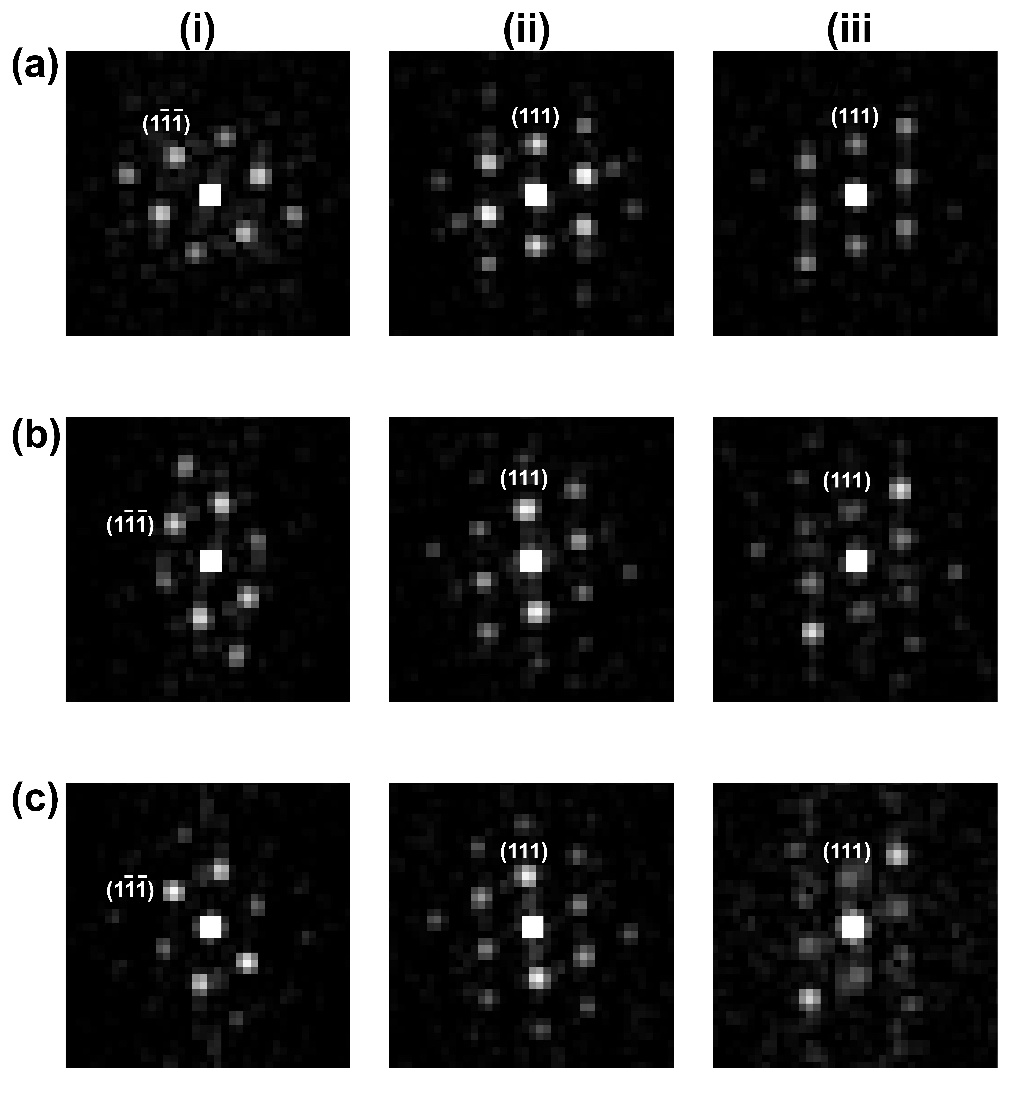


**Figure S12.** The FFT patterns collected from ETEM in Figure 4 (three regions (i to iii): upper-left *c*-region (i), middle *c*-phase region (ii), and lower-right *c*-/*a*-region (iii)) at different time: 3.72 min (a), 21.58 min (b), and 22.15 min (c).


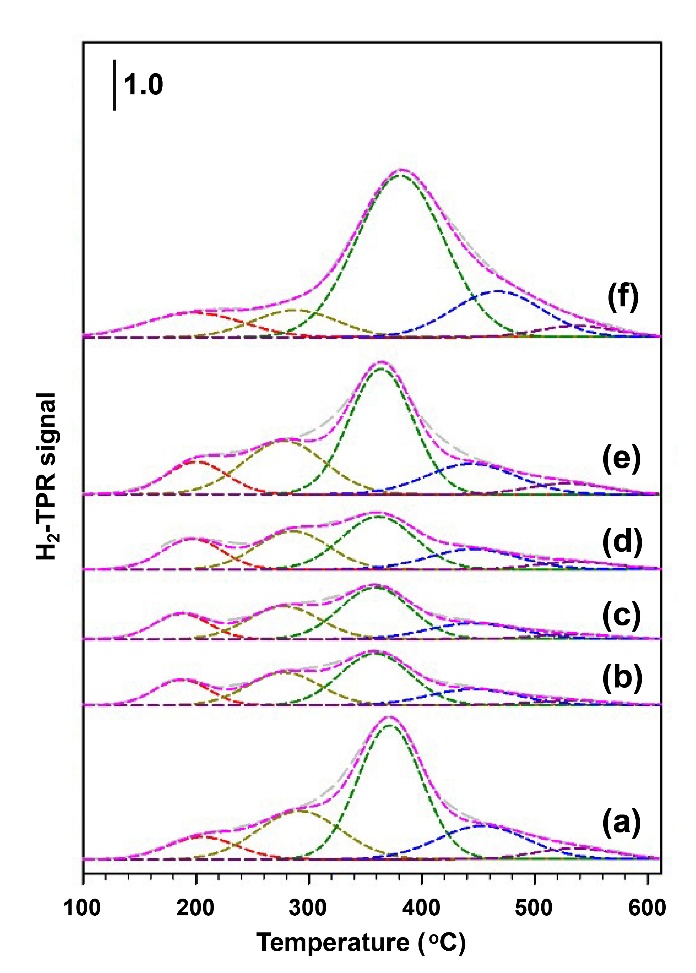


**Figure S13.** H_2_-TPR curves of CuAu/Al_2_O_3_ after treatments under O_2_ at different oxygenation temperatures, (a) 50 ^o^C, (b) 100 ^o^C, (c) 150 ^o^C after 400 ^o^C, (d) 200 ^o^C, (e) 300 ^o^C after 50 ^o^C, (f) 400 ^o^C. Color lines: deconvoluted peaks.


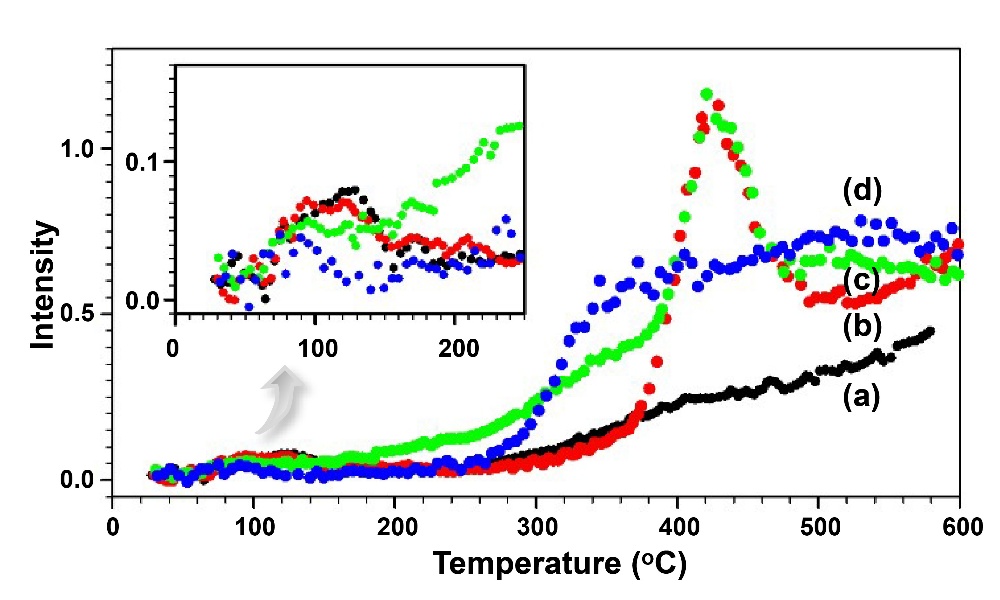


**Figure S14.** CO TPR results of Cu_50_Au_50_(N_2_)/Al_2_O_3_ catalysts after pre-oxidized under (a) no oxidation; (b) 5 mins ramping to 400 ^o^C + 5 mins soak at 400 ^o^C; (c) 5 mins ramping to 400 ^o^C + 20 mins soak at 400 ^o^C, and (d) 5 mins ramping to 400 ^o^C + 60 mins soak at 400 ^o^C. Inset: a zoomed view of the lower temperature region.


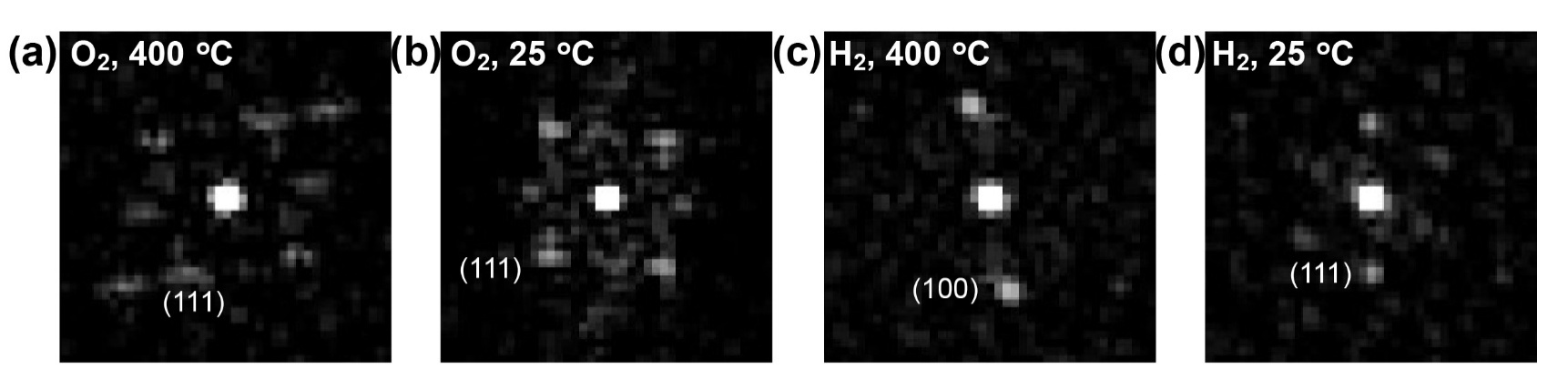


**Figure S15.** The FFT patterns collected from ETEM in Figure 5(a-b) under O_2_ (a-b) and H_2_ (c-d) at 400 ^o^C and 25 ^o^C, respectively.


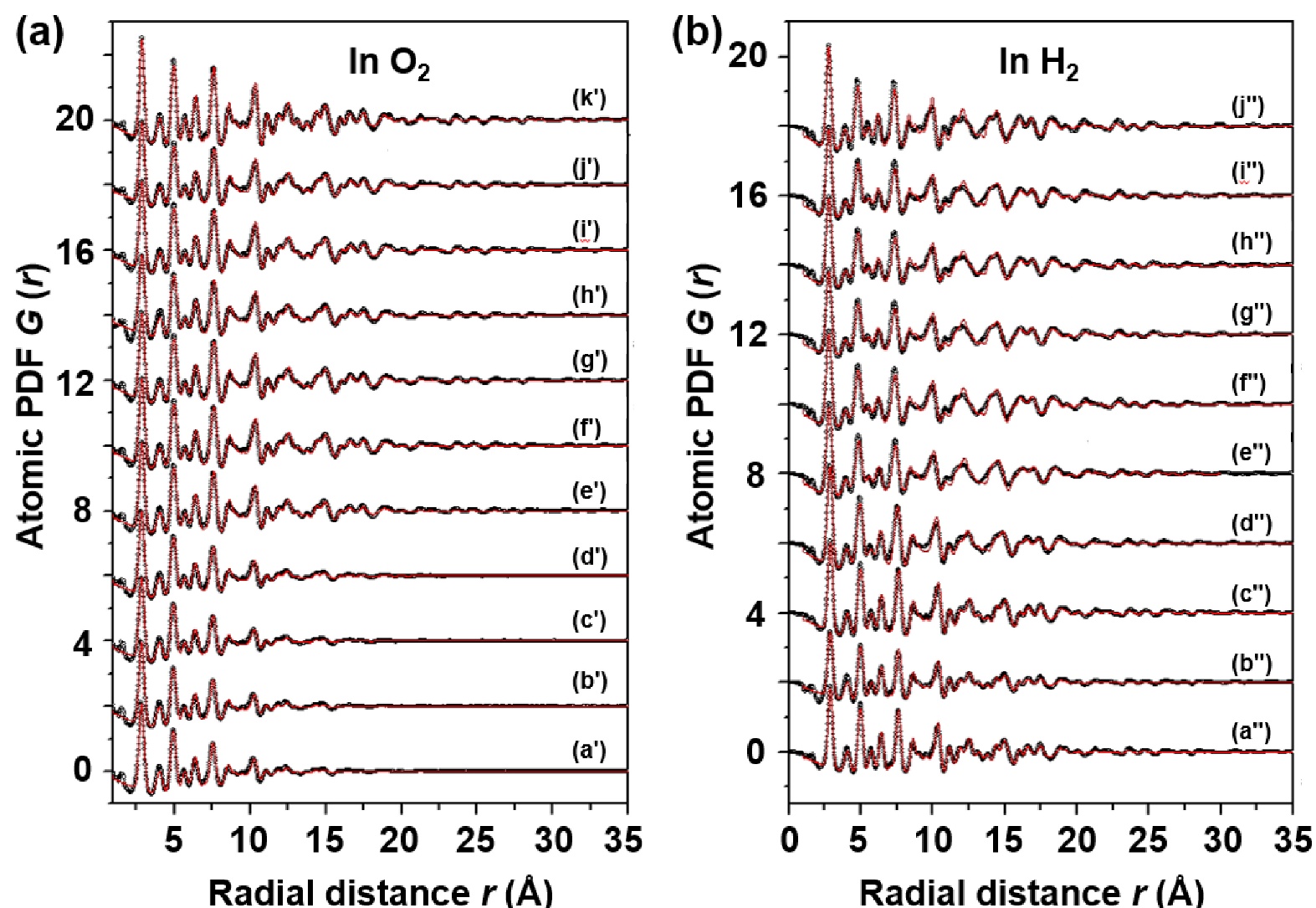


**Figure S16.** In-situ HE-XRD/PDF data. Atomic PDFs for Au_51_Cu_49_/C obtained from in-situ HE-XRD data collected under first 10 vol.% O_2_ (a), followed by 5 vol.% H_2_ (b). Experimental data – symbols, computed data – red solid line (based on an fcc-type structure). (a): (a') under room *T*, *a*=4.04576 Å; (b') under 50 ^o^C, *a*=4.04824 Å; (c') under 100 ^o^C, *a*=4.05225 Å; (d') under 150 ^o^C, *a*=4.05752 Å; (e') under 200 ^o^C, *a*=4.0703 Å; (f') under 260 ^o^C/ 5 min, *a*=4.08094 Å; (g') under 260 ^o^C/ 10 min, *a*=4.08185 Å; (h') under 260 ^o^C/ 20 min, *a*=4.08355 Å; (i') under 260 ^o^C/ 30 min, *a*=4.08367 Å; (j') under 260 ^o^C/ 60 min, *a*=4.08457 Å; (k') back to room *T*, *a*=4.07056 Å. (b): (a'') pretreated in O_2_, *a*=4.07056 Å; (b'') under 50 ^o^C, *a*=4.07475 Å; (c'') under 150 ^o^C, *a*=4.079 Å; (d'') under 200 ^o^C, *a*=4.0415 Å; (e'') under 250 ^o^C, *a*=3.96481 Å; (f'') under 300 ^o^C/ 5 min, *a*=3.94297 Å; (g'') under 300 ^o^C/ 10 min, *a*=3.94213 Å; (h'') under 300 ^o^C/ 20 min, *a*=3.94111 Å; (i'') under 300 ^o^C/ 30 min, *a*=3.93998 Å; (j'') back to room *T*, *a*=3.92251 Å.


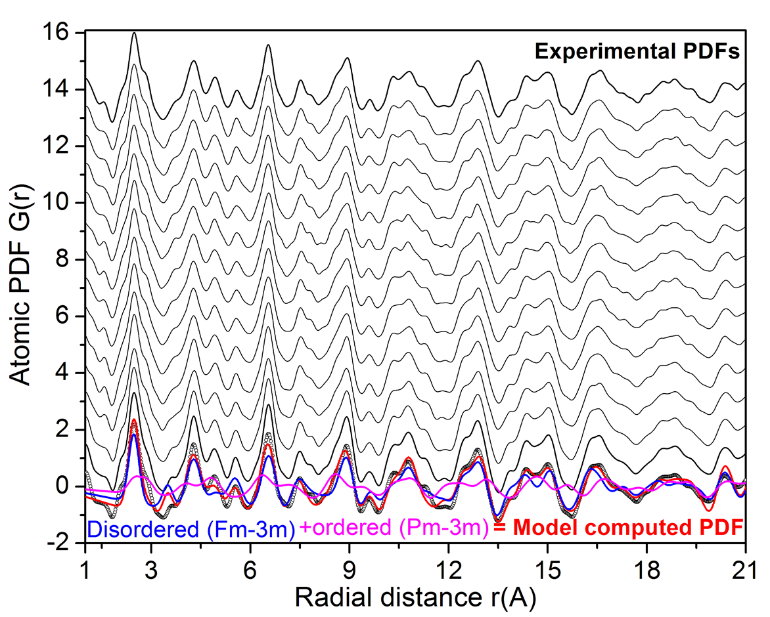


**Figure S17.** In-situ HE-XRD derived atomic PDFs and their model computed PDFs for Al_2_O_3_ supported Cu_50_Au_50_ (N_2_) nanoalloys during processes including heating under helium from room temperature to 150 ^o^C, isothermal process under CO + O_2_ reaction atmosphere, and cooling down to room temperature. (original atomic PDFs: black dots, model computed PDFs: red curve; disordered fcc structure: blue curve; and ordered fcc structure: pink curve).


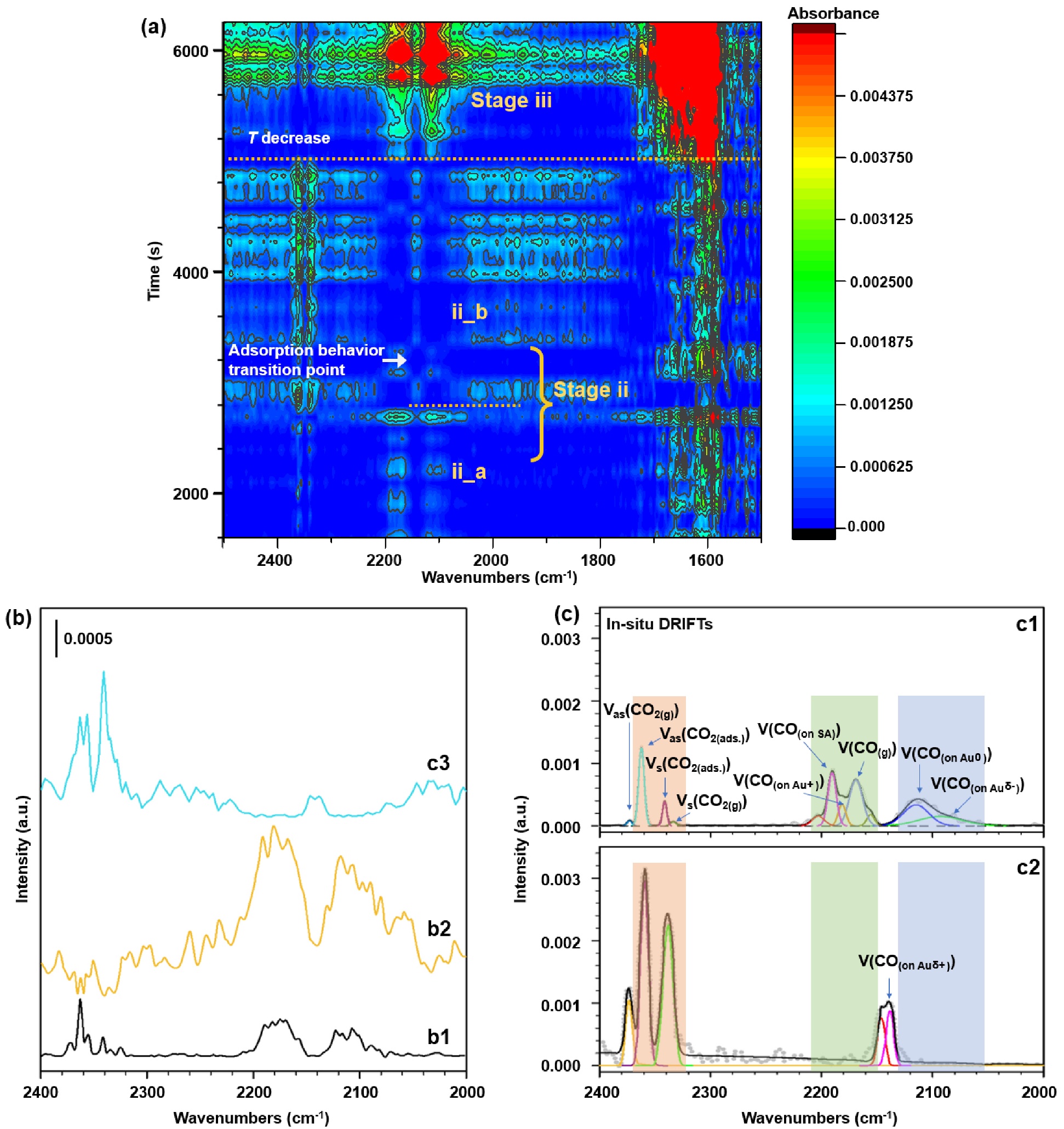


**Figure S18.** (a) 2D-contour plot of time-resolved in-situ DRIFTs spectra for Al_2_O_3_ supported Cu_50_Au_50_ (N_2_) nanoparticles during processes including heating under helium from room temperature to 150 ^o^C, isothermal process under CO + O_2_ reaction atmosphere, and cooling down to room temperature. (b-c) Snapshots of in-situ DRIFTs spectra at different times (b: b1, 300 s; b2, 1300 s, b3, 2100 s; c: c1, 600 s; c2, 2600 s,), showing peak characteristics in adsorbed CO_2_ and CO regions (highlighted in light blue (low-ν), green (middle-ν), and orange (high-ν)).


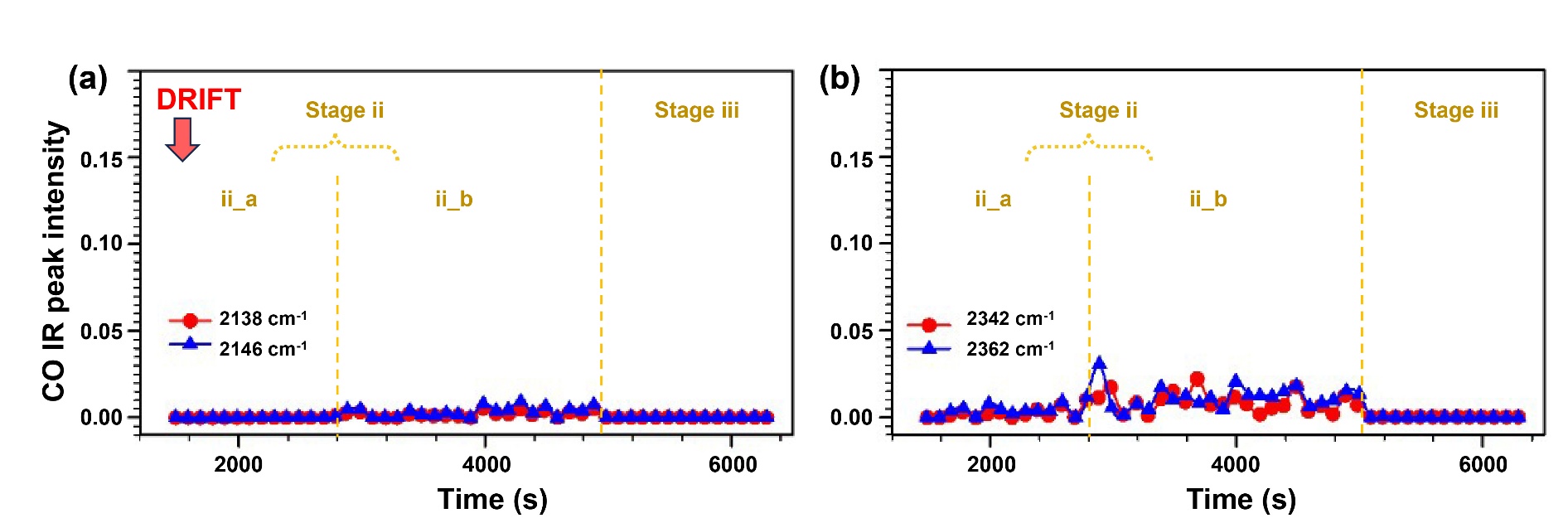


**Figure S19.** Plots of CO peak intensities vs. time. (a-b) ~ 2140 cm^-1^ (middle frequency) associated with CO linearly adsorbed on Au^δ+^ (a); and ~ 2350 cm^-1^ (high frequency) associated with CO_2_ linearly adsorbed on Au^δ++^ (b). Time-resolved in-situ DRIFT spectra starts from (i) heating under He to 150 ^o^C, (ii) CO+ O_2_ reaction at 150 ^o^C, and (iii) cooling down to room temperature.


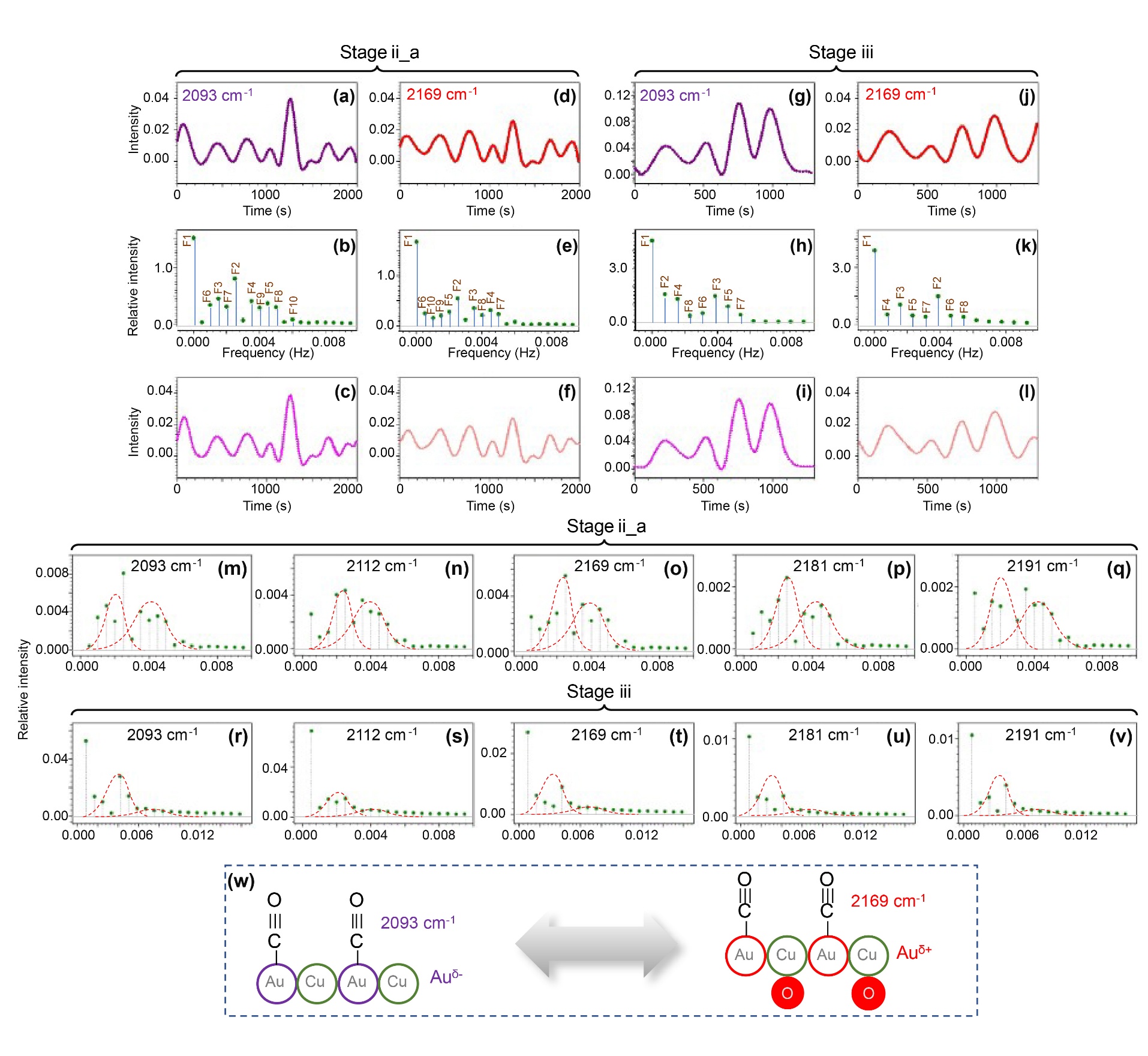


**Figure S20**. FFT (Fast-Fourier Transform) and HHT (Hilbert–Huang Transform) analyses of the DRIFTs irregular oscillation patterns. (a, d, g, j) Results from FFT analysis of the DRIFTs irregular oscillation patterns: (b, e, h, k): experimental data; (b-k): FFT simulation of the data generating the frequencies (F1, 2, etc.); and (c, f, i, l) the oscillation patterns simulated by sum of F2 to F8 frequencies. (m-v) Comparison of the results from the FFT with those from the from IMF1 and 2 derived from HHT analysis of the original signal (dashed red curves) showing the frequency distributions (IMF1 and IMF2). The frequency distributions are derived from HHT analysis (see Supporting Information). (w) A schematic illustration showing two possible oscillatory states of the surface-active sites for the adsorption and desorption of CO or CO_2_ on Au^δ+^ (with O-species on Cu atoms) and Au^δ-^ sites (without O-species on Cu atoms) during surface reaction under the reaction condition.


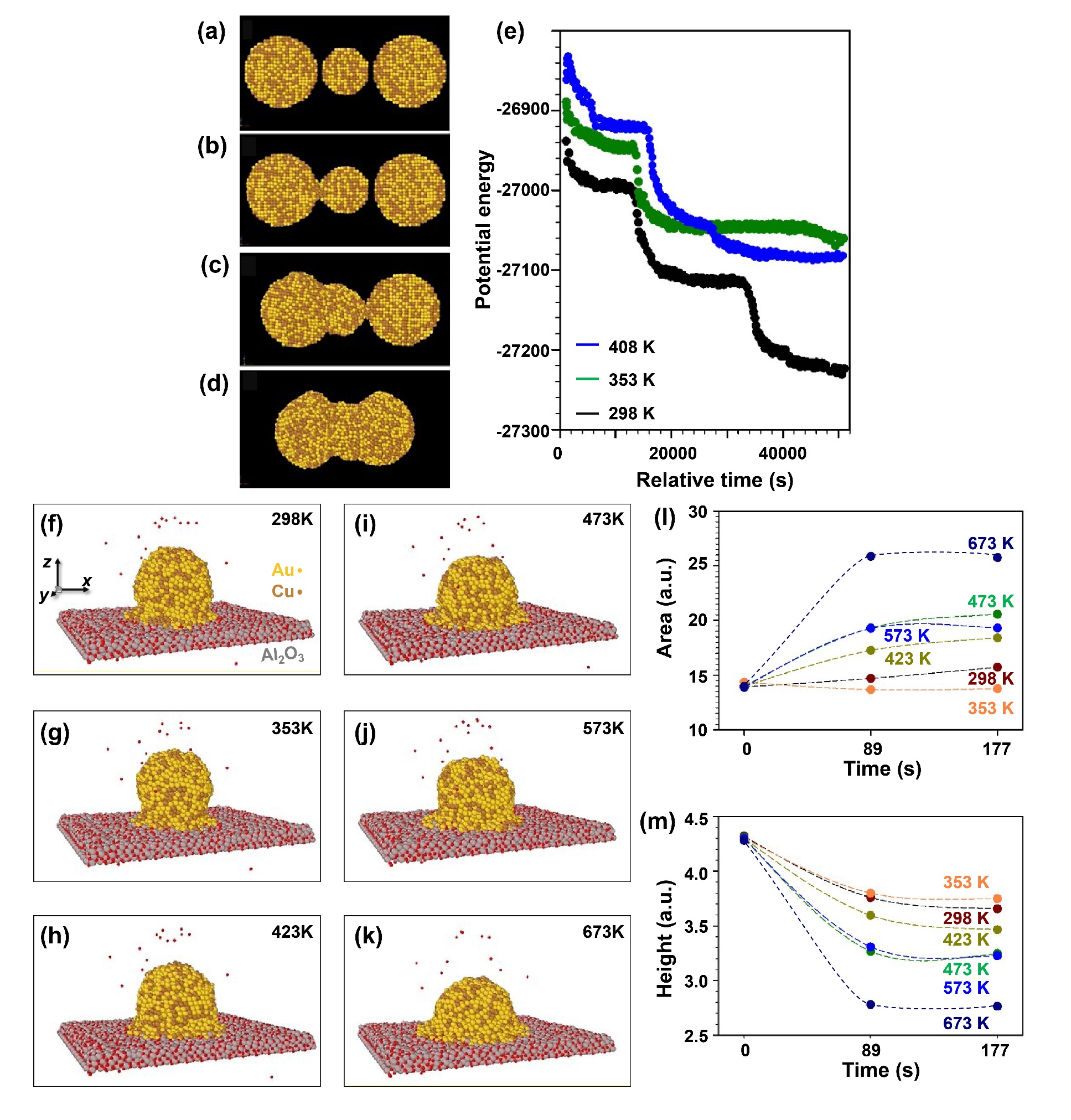


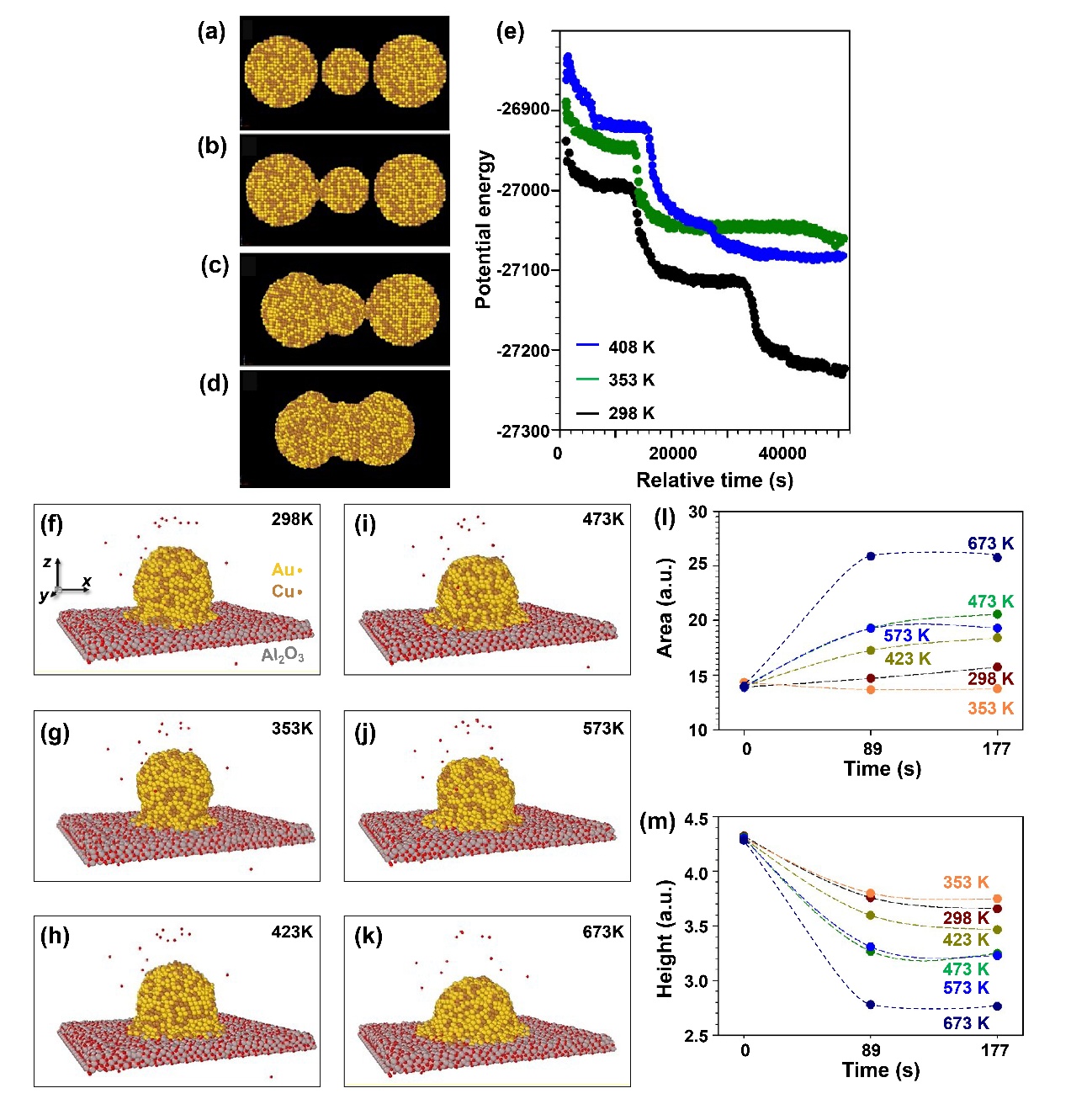


**Figure S21**. (a-e) Preliminary MD simulations. Snapshots (a-d) and potential energy curves (e) for unsupported CuAu nanoparticles sintering at different temperatures. Time sequence: from (a) to (d). Model: a 3-NP model of CuAu nanoparticles in which the smaller sized NP (3 nm) is located in between the larger-sized NPs (4 nm). (f-m) Snapshots (f-k) and evolution of Al_2_O_3_ supported CuAu NP’s contact area (l) and height (m) with the Al_2_O_3_ substrate vs. time at different temperatures. Note: time is relative time.

**Reference**

[79] P. N. Njoki, I. I. S. Lim, D. Mott, H. Y. Park, B. Khan, S. Mishra, R. Sujakumar, J. Luo, C. J. Zhong, *J Phys Chem C* **2007**, *111*, 14664-14669.

[80] P. N. Njoki, J. Luo, M. M. Kamundi, S. Lim, C. J. Zhong, *Langmuir* **2010**, *26*, 13622-13629.

[81] D. Mott, N. T. B. Thuy, Y. Aoki, S. Maenosono, *Philos T R Soc A* **2010**, *368*, 4275-4292.

[82] S. Nishimura, D. Mott, A. Takagaki, S. Maenosono, K. Ebitani, *Phys Chem* *Chem Phys* **2011**, *13*, 9335-9343.

[83] B. R. Chen, L. A. Crosby, C. George, R. M. Kennedy, N. M. Schweitzer, J. G. Wen, R. P. Van Duyne, P. C. Stair, K. R. Poeppelmeier, L. D. Marks, M. J. Bedzyk, *ACS Catal* **2018**, *8*, 4751-4760.

[84] Y. L. Liu, A. R. H. Walker, *Angew Chem Int Edit* **2010**, *49*, 6781-6785.

[85] X. M. Lian, Principles of Dynamic Signal Inner Product Transformation, Tsinghua University Press, China, **2016**, p. 39. https://www.tup.com.cn/bookscenter/book_06840301.html

[86] N. E. Huang, Z. Shen, S. R. Long, M. C. Wu, H. H. Shih, Q. Zheng, N. C. Yen, C. C. Tung, H. H. Liu, *Proc. R. Soc. Lond. A* **1998**, *454*, 903-995.

[87] Y. Niu, X. Huang, Y. Wang, M. Xu, J. Chen, S. Xu, M.-G. Willinger, W. Zhang, M. Wei, B. Zhang. Manipulating interstitial carbon atoms in the nickel octahedral site for highly efficient hydrogenation of alkyne. *Nat. Commun*. **2020,** *11*, 3324.

[88] X. Sun, W. Zhu, D. Wu, C. Li, J. Wang, Y. Zhu, X. Chen, J. A. Boscoboinik, R. Sharma, G. Zhou. Surface-reaction induced structural oscillations in the subsurface. *Nat. Commun*. **2020**, *11*, 305.
